# Over 50,000 Metagenomically Assembled Draft Genomes for the Human Oral Microbiome Reveal New Taxa

**DOI:** 10.1016/j.gpb.2021.05.001

**Published:** 2021-09-04

**Authors:** Jie Zhu, Liu Tian, Peishan Chen, Mo Han, Liju Song, Xin Tong, Xiaohuan Sun, Fangming Yang, Zhipeng Lin, Xing Liu, Chuan Liu, Xiaohan Wang, Yuxiang Lin, Kaiye Cai, Yong Hou, Xun Xu, Huanming Yang, Jian Wang, Karsten Kristiansen, Liang Xiao, Tao Zhang, Huijue Jia, Zhuye Jie

**Affiliations:** 1 BGI-Shenzhen, Shenzhen 518083, China; 2 Shenzhen Key Laboratory of Human Commensal Microorganisms and Health Research, BGI-Shenzhen, Shenzhen 518083, China; 3 Shenzhen Engineering Laboratory of Detection and Intervention of Human Intestinal Microbiome, BGI-Shenzhen, Shenzhen 518083, China; 4 Laboratory of Genomics and Molecular Biomedicine, Department of Biology, University of Copenhagen, Copenhagen DK-2100, Denmark; 5 James D. Watson Institute of Genome Sciences, Hangzhou 310058, China; 6 BGI-Qingdao, BGI-Shenzhen, Qingdao 266555, China

**Keywords:** Metagenomics, Human oral microbiome, Metagenome-assembly genome, Genome catalog, Gender

## Abstract

The oral cavity of each person is home to hundreds of bacterial species. While taxa for oral diseases have been studied using culture-based characterization as well as amplicon sequencing, metagenomic and genomic information remains scarce compared to the fecal microbiome. Here, using metagenomic shotgun data for 3346 oral **metagenomic** samples together with 808 published samples, we obtain 56,213 metagenome-assembled genomes (MAGs), and more than 64% of the 3589 species-level genome bins (SGBs) contain no publicly available genomes. The resulting genome collection is representative of samples around the world and contains many genomes from candidate phyla radiation (CPR) that lack monoculture. Also, it enables the discovery of new taxa such as a genus *Candidatus* Bgiplasma within the family Acholeplasmataceae. Large-scale metagenomic data from massive samples also allow the assembly of strains from important oral taxa such as *Porphyromonas* and *Neisseria*. The oral microbes encode genes that could potentially metabolize drugs. Apart from these findings, a strongly male-enriched *Campylobacter* species was identified. Oral samples would be more user-friendly collected than fecal samples and have the potential for disease diagnosis. Thus, these data lay down a genomic framework for future inquiries of the **human oral microbiome**.

## Introduction

The human microbiome has been implicated in a growing number of diseases. The majority of microbial cells are believed to reside in the large intestine [1] and cohorts with fecal metagenomic data contain over 1000 individuals [2], [3].

For the oral microbiome, culture-based characterization as well as marker gene sequencing techniques has been applied in many oral bacteria-associated disease studies, such as cystic fibrosis with *Streptococcus oralis* [4], colorectal cancer (CRC) with *Fusobacterium nucleatum* [5], and even Alzheimer’s disease with *Porphyromonas gingivalis* [6]. With the ultra-fast growing culture-independent next generation sequencing technologies, hundreds of metagenomic shotgun-sequenced samples have been available from the Human Microbiome Project (HMP) and for the research of rheumatoid arthritis [1], [7], [8]. Several other diseases studied by accurate and robust metagenome-wide association studies (MWAS) using gut microbiome data indicated potential contributions from the oral microbiome in disease etiology [9], [10], [11], [12], [13], [14]. Although the MWAS on rheumatoid arthritis was based on a *de novo* assembled reference gene catalog for the oral microbiome [8], analyses on genomes would be more desirable. For diagnosis of some diseases, gathering oral samples shows advantages like higher sensitivity and accuracy, better prognosis, and better patient satisfaction than fecal samples. Moreover, collecting oral samples is more convenient as it is easy to operate and could be taken at a fully controlled setting witnessed by trained professionals. Unlike the anaerobic environment for the gut microbiome, the oral microbiome is believed to be well covered by culturing [15], and the analyses by 16S rRNA gene sequencing or PCR are common.

Recent published large-scale metagenomic assembly efforts mostly included fecal metagenomic data [16], [17], [18]. It is not clear how much is missing for the oral microbiome. The most well-characterized oral microbial database, the expanded Human Oral Microbiome Database (eHOMD), has about 750 oral taxa; however, only 57% of the oral bacterial species have been officially named, 13% have been cultivated yet remain unnamed, and 30% are uncultivated [19]. A representative genome catalog for genome-resolved analysis is strongly required.

In this study, we present 3346 new oral metagenomic samples. A total of 56,213 medium- and high-quality metagenome-assembled genomes (MAGs) are constructed based on our collection together with previously published 808 samples. The 56,213 MAGs together with 190,309 public genomes were clustered into 3589 oral species-level clades. New taxa as well as the substantially complemented genomic content of known species are revealed. We provide a genome reference that is highly representative of metagenomic samples not used in assembly and could facilitate culturing and functional screens, as well as disease diagnosis and modulation based on the oral microbiome.

## Results

### Draft genomes assembled from oral metagenomic data

**Figure 1:**
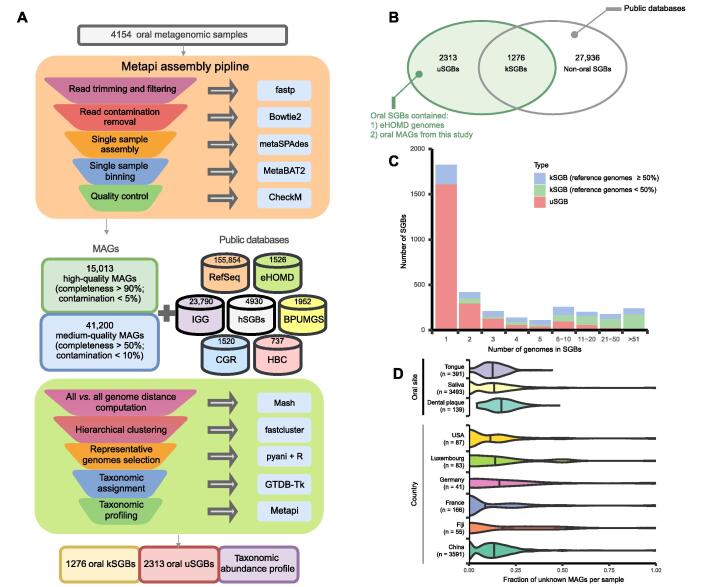
**Atotalof3589 oral SGBs assembled from 4154 (3346 new samples) meta-analyzed oral metagenomes** **A.** The workflow for SGB construction. **B.** Reconstructed MAGs and genomes from public databases were clustered into 31,525 SGBs based on ANI. A total of 3589 oral SGBs (green background) which contained eHOMD genomes or oral MAGs from this study were divided into 1276 kSGBs and 2313 uSGBs. The kSGBs contained pubilc genomes, and the uSGBs only consisted of MAGs from this study. **C.** Genome number distribution of uSGBs and kSGBs. **D.** Distribution of fraction of unknown MAGs in each sample from different oral sites and geographic origins. Numbers in brackets indicate the sample size. SGB, species-level genome bin; kSGB, known SGB; uSGB, unknown SGB; MAG, metagenome-assembled genome; ANI, average nucleotide identity; eHOMD, the expanded Human Oral Microbiome Database.

We performed shotgun sequencing on 2284 saliva and 391 tongue dorsum samples from the 4D-SZ cohort (trans-omics, with more time points in future studies, based in China) [2], [11], [20], and 671 saliva samples from five ethnic groups from Yunnan Province, China. Over 43.19 terabytes of sequence data were generated. Together with 808 published oral samples from 5 studies [8], [21], [22], [23], [24] that have not been used in a recent large-scale assembly study [18], a total of 4154 oral samples with metagenomic data were obtained (Table S1). A single sample assembly, single sample binning strategy was used. In brief, the metagenomic shotgun reads were assembled into contigs using metaSPAdes [25] independently. Contigs longer than 1500 bp (average 14,094 contigs per sample) were binned by MetaBAT2 [26] in each sample, leading to 163,718 MAGs. After quality control by CheckM [27], 56,213 MAGs which agreed the medium-quality standards [28] (> 50% completeness, < 10% contamination) were retained for further analysis (Figure 1A; Table S2). Of these, 15,013 MAGs reached the standards for high-quality (> 90% completeness, < 5% contamination). Moreover, 73.25% of high-quality MAGs and 26.57% of medium-quality MAGs had at least 18 types of standard 20 amino acids decoded by tRNAs (Figure S1A). All three (23S, 16S, and 5S) rRNA genes were present in 27.28% of high-quality MAGs and 13.13% of medium-quality MAGs (Figure S1B).

### New genomes from the new samples

To assess the novelty of our assembled genomes, we comprehensively incorporated 190,309 existing isolate and metagenome-assembled genomes from NCBI RefSeq, eHOMD [29], and recent publications [16], [17], [18], [30], [31] (Figure 1A; Table S1) with reconstructed MAGs in this study. A total of 246,522 genomes were obtained.

Species-level genome bins (SGBs) were computed for the 246,522 genomes following multiple steps (Figure 1A, see Methods and methods for details), defined as at least 95% average nucleotide identity (ANI) and at least 30% overlap of the aligned genomes. The clustering analysis resulted in 31,525 SGBs. Among them, 3589 SGBs with oral reference genomes from eHOMD or oral MAGs were defined as oral SGBs (Figure 1B). In the oral SGBs, 2313 clusters (64% of the total oral species) only contained MAGs from this study (denoted uSGBs for unknown SGBs), some of which were repeatedly captured in our data, with more than 50 genomes each (Figure 1C); the 1276 known oral SGBs (kSGBs) could be further divided according to the percentage of reference genomes in the cluster. Interestingly, kSGBs with < 50% reference genomes outnumbered kSGBs with ≥ 50% reference genomes for clusters containing more than 10 genomes (Figure 1C), underscoring the discovery power of large metagenomic cohorts. MAGs assembled from the 4D-SZ cohort made the greatest contribution to the number of uSGBs, followed by the rheumatoid arthritis cohort and the Yunnan cohort (Figure S2A). The number of medium- and high-quality MAGs per sample increased with the microbial sequence bases, reaching saturation at 15 gigabases (Figure S2B). Regarding the ratio of new MAGs in the samples, we retrieved a greater fraction of previously unknown genomes in dental samples than in saliva or tongue samples, even though we had many more saliva samples than tongue samples and published dental samples (Figure 1D). This ratio also appeared to differ between cohorts, with less than 10% unknown MAGs for samples from France or USA, and more newly matched uSGBs for samples from Fiji, Germany, and Luxembourg (Figure 1D). The large cohort available from this study is crucial for the retrieval of novel oral species, contributing over 2000 uSGBs, which greatly expands our knowledge of oral microbiome diversity.

### Close to 90% representation of oral metagenomic data by the genomes

**Figure 2:**
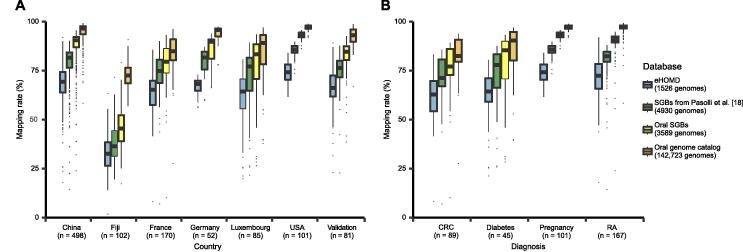
**The expanded genome set substantially increases the mappability of oral metagenomes** Reads from all the 808 public samples, 100 randomly selected samples from Shenzhen and Yunnan in China, and 81 additional verified samples which haven’t been assembled were mapped against databases including eHOMD, representative genomes of human SGBs from Pasolli et al. [18], representative genomes of oral SGBs, and all genomes from oral genome catalog. **A.** Mappability of samples from different country and a verified data set. **B.** Mappability of the public samples with diseases as well as pregnancy. The numbers in brackets under the boxes indicate the sample size. CRC, colorectal cancer; RA, rheumatoid arthritis.

We next examined the ability of this species-level genome set to represent the metagenomic shotgun data. We assessed the percentage of reads that could align to cultured genomes (eHOMD) only and cultured complemented by metagenomically assembled genomes (Table S3). The 1526 genomes from eHOMD led to a median mapping rate of 67.86%; the 4930 representative human SGBs from a recent large-scale assembly study [18] led to a median mapping rate of 80.12%; the 3589 representative oral SGBs from the current study led to a median mapping rate of 88.29%, especially for metagenomes from USA and Germany; and the 81 saliva and subgingival metagenomes from three verified cohorts [32], [33], [34] that were not used in the assembly process even led to a median mapping rate of 84.67% (Figure 2A; Table S1). Across physiological states, our SGBs well represented pregnant samples from USA (reaching a median mapping rate of 93.22%), rheumatic arthritis (RA) samples (reaching a median mapping rate of 91.00%), and diabetes samples (reaching a median mapping rate of 85.49%) (Figure 2B). Such a high degree of representation of metagenomic data across geography, ethnicity, age, and physiological states suggests that the expanded genomic content of oral SGBs could serve as a starting point for quantitative taxonomic and functional analyses of the human oral microbiome.

While with all genomes from the oral genome set, the median mapping rate rose to 94.36%. The remaining unmapped reads were mainly classified as homo using Kraken2 [35], especially for metagenomes from France and Luxembourg (Figure S3A). For Fiji, besides homo contamination, 12.07% of unmapped reads were classified as bacteria, and 57.40% of unmapped reads were still unclassified (Figure S3B).

### Taxonomic landscape of the oral microbial genomes

**Figure 3:**
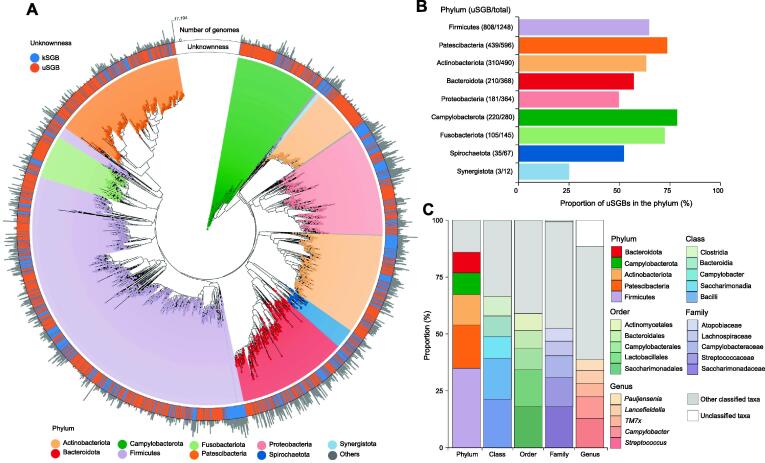
**Phylogeny of representative oral SGBs** **A.** Oral-associated microbial phylogenetic tree of representative genomes from SGBs. Clades and their backgrounds are colored based on the phylum level. Unknownness of SGBs is shown in the ring. Outer histogram with log_10_ axis shows the genome numbers of SGBs. **B.** Proportion of uSGBs at the phylum level. Numbers of uSGBs and total SGBs are shown in brackets. **C.** Taxonomic composition of the 2313 uSGBs compared to GTDB. Five most frequently observed taxa at the phylum, class, order, famlily, and genus levels are shown in different colors; unclassified taxa are filled in white; and the remaining lineages are grouped as “other classified taxa” and shown in gray. GTDB, Genome Taxonomy Database.

The taxonomic classification of 3589 representative genomes of oral SGBs was assigned using GTDB-Tk [36], [37], [38], [39], [40] with external Genome Taxonomy Database (GTDB) release 89. Similar to the gut microbiome, Firmicutes took up the largest number of branches (1248 SGBs, 44,217 genomes). The other SGBs distributed into 15 phyla, including major human oral phyla such as Actinobacteria (490 SGBs, 14,574 genomes), Bacteroidetes (368 SGBs, 23,976 genomes), Proteobacteria (364 SGBs, 49,270 genomes), Campylobacterota (280 SGBs, 3295 genomes), and Fusobacteriota (145 SGBs, 2124 genomes) (Figure 3A; Table S3). uSGBs accounted for 64.45% in the total reconstructed phylogenetic branch length, with 78.57% of the diversity in the Campylobacterota phylum contributed by the new uSGBs, followed by 73.66% for Patescibacteria and 72.41% for Fusobacteriota (Figure 3B), which seemed overlooked by culturing studies. We estimated that there was the median of 210 SGBs with a relative abundance higher than 0.1% per sample (Figure S2C). Besides, uSGB also had very high abundance, explained for 68.10% of richness and 65.23% of relative abundance per sample (Figure S2D and E). Our MAGs greatly expanded the species’ or strains’ diversity within each phylum. As many as 596 SGBs from 4028 genomes belonged to the candidate superphylum of Patescibacteria (Parcubacteria, also known as OD1), which only had 157 kSGBs with 178 reference genomes. We noted a few not so well studied phyla that were interesting in analogy to the gut microbiome. *Akkermansia* is the only genus from the Verrucomicrobiota phylum in the human gut and intensively pursued for its role in health and diseases, and the Verrucomicrobiota and Spirochaetota phyla take up a greater fraction in Hadza hunter gatherers compared to developed countries [41]. Here, we identified 67 SGBs from 958 genomes belonging to the Spirochaetota phylum, while only 32 kSGBs with 121 reference genomes were available for Spirochaetota. Moreover, we identifed 142 kSGBs with 1526 genomes belonging to the Patescibacteria phylum (candidate division Saccharibacteria,TM7) (Table S3).

In addition, 265 of the 2313 uSGBs had taxonomic information until order or family level, but cannot be annotated to a known genus. The top 3 uSGB classified families were Saccharimonadaceae (17.99%), Streptococcaceae (12.88%), and Campylobacteraceae (9.51%), whereas the top 3 assigned genera were *Streptococcus* (12.88%), *Campylobacter* (7.65%), and *TM7x* (5.92%) (Figure 3C).

### A new genus with small genomes

**Figure 4:**
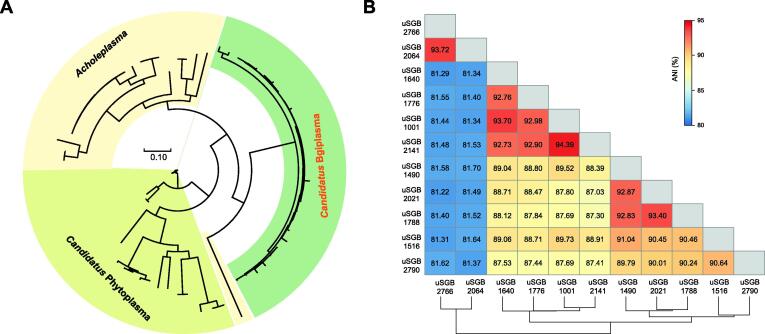
**A new candidatusgenusis found within the Acholeplasmataceaefamily** **A.** Phylogenetic tree drawn from all MAGs in the new candidatus genus *Candidatus* Bgiplasma and public genomes in the Acholeplasmataceae family. **B.** ANI among all uSGBs in the *Candidatus* Bgiplasma genus indicates two species clades (ANI < 85%).

In the Acholeplasmataceae family of the Bacilli class, a number of our uSGBs with high-quality MAGs formed a clade with shallow branches, which was distinct from the *Acholeplasma* and *Candidatus* Phytoplasma genera (Figure 4A). The genome size of this genome-defined genus (temporarily denoted as *Candidatus* Bgiplasma) was 0.69 ± 0.05 Mb, which was similar to that of *Candidatus* Phytoplasma (0.64 ± 0.14 Mb), but much smaller than that of *Acholeplasma* (1.50 ± 0.20 Mb). However, the genome of *Candidatus* Bgiplasma was complete according to single-copy marker genes in CheckM (Table S2). The GC contents of the three clades were also different. The *Candidatus* Bgiplasma genus was more toward normal GC content (34.57% ± 0.21%), but not as low as those of *Acholeplasma* (30.99% ± 1.75%) and *Candidatus* Phytoplasma (25.98% ± 2.68%) (Table S5). Despite the lack of deep branches, the ANI distribution of uSGBs within the *Candidatus* Bgiplasma genus showed two separate groups at species-level divergence (ANI < 85%) (Figure 4B), illustrating diversity within this new genus. These 11 uSGBs comprising 29 MAGs contributed more than 0.1% relative abundance in 209 of 4154 (5.03%) samples, indicating that this genus is a potentially important but so far uncharacterized clade in the oral microbiome.

All genes of the *Candidatus* Bgiplasma genus were annotated by eggNOG mapper [42], [43] and the rate of annotation was 81.83% (Table S5). Within the compact genome of *Candidatus* Bgiplasma, Clusters of Orthologous Groups (COG) categories of replication, recombination and repair, posttranslational modification, protein turnover, chaperones, and inorganic ion transport and metabolism are found (Figure S4).

### Distribution of species and strains

**Figure 5:**
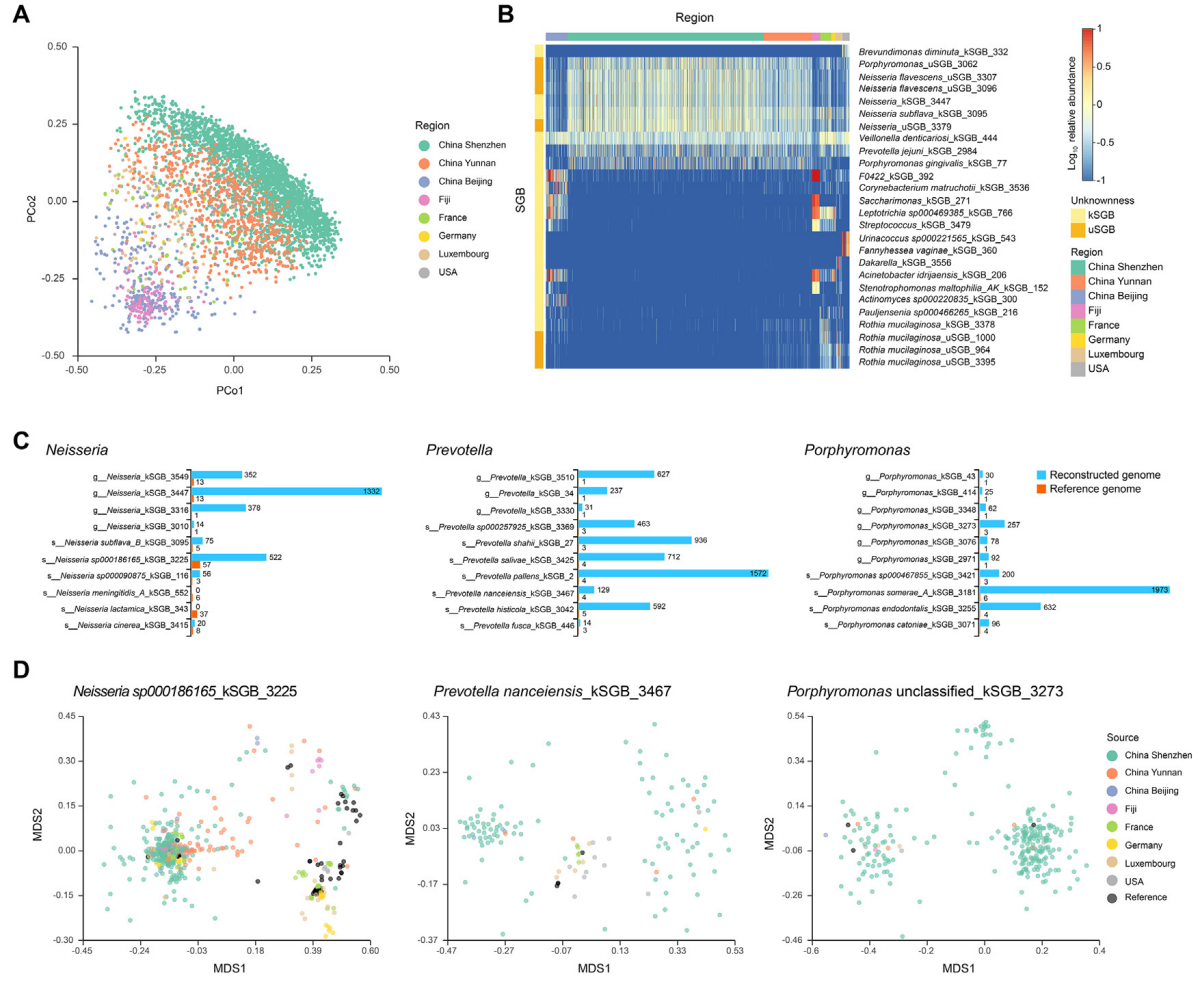
**Geographic distribution of oral SGBs and strains** **A.** PCoA plot based on Bray-Curtis distances of oral SGB relative abundance profile highlights distinct microbial communities among different original populations. **B.** Heatmap of top 5 abundant SGBs in each geographic region. Columns are samples from different regions separated by colors. Unknownness and classification of top SGBs are shown on the left and right of the heatmap, respectively. **C.** Bar plot showing genome numbers of top 10 most frequent species from common oral genus. Reconstructed MAGs largely extend the reference set. **D.** MDS showing the distribution of assembled MAGs and reference genomes based on ANI. Only high-quality MAGs and reference genomes are presented here. Points are genomes colored by country. PCoA, principal coordinate analysis; MDS, multidimensional scaling.

The new samples from this study differed in oral microbiome composition compared to published samples across geography (Figure 5A). Three groups of Chinese samples (Shenzhen: southern China, Yunnan: southwest China, Beijing: northern China) appeared to be as distinct from each other as those from any of the other geographic groups. Both the 4D-SZ and Yunnan samples abundantly contained many uSGBs, such as *Neisseria flavescens*, *Neisseria* spp., and *Porphyromonas* spp., and kSGBs, such as *Porphyromonas gingivalis* (an opportunistic oral pathogen), which was rare in the other cohorts (Figure 5B). Pregnant samples from USA contained *Fannyhessea vaginae* (the vaginal pathogen previously known as *Atopobium vaginae* [44]), *Urinacoccus*, *etc.*, which were of much lower abundance in other cohorts (Figure 5B). Samples from Fiji, although not well mapped (Figure 2A), showed high levels of a few SGBs that were also seen in the RA study from Beijing, China, including an SGB from *Saccharimonas* (Figure 5B).

At the strain level, the new samples from the current study greatly expanded the genome collection for common taxa such as *Neisseria* spp., *Porphyromonas* spp., and *Prevotella* spp. (Figure 5C and D). The numbers of publicly available reference genomes for the top 10 most abundant species in the genera *Porphyromonas* and *Prevotella* were less than 10, and less than 100 for the genus *Neisseria*. Here we obtained more than 1000 genomes for a few of the species and increased the diversity in all the species in these genera (Figure 5C). And rarefaction analyses revealed that the number of genes in both the public reference genome and our reconstructed MAGs was significantly higher than the public reference genome only (Figure S5). The phylogenetic structure of select species with a large number of genomes also suggests strain-level variations (subspecies). The *Prevotella nanceiensis*_kSGB_3467, for example, included 3 reference genomes that were similar to a few genomes from developed countries, while our samples contributed two large clusters that were more distantly related (Figure 5D).

### Potential functions in drug metabolism

Many human-targeted drugs are reported to be metabolized to their inactive form by the human gut microbiome or to be affected by the gut bacteria [45], [46], [47]. We conducted literature survey and curated experiments which confirmed that microbiome genes could metabolize drugs. To address the potential of drug metabolite of oral microbiome, we mapped genes that could metabolize drugs to our oral SGB genomic contents. Interestingly, we found that many oral communities contained homologs to those gut bacteria encoding enzymes relating up to 41 human-targeted drugs (Table S6). With the same method, we also identified 20 genes that encode key enzymes involved in 12 human diseases, and 6 genes that produce non-traditional antibacterial therapeutic compounds in our oral SGB genomic contents (Table S6). These results suggest that the oral microbiome may play an important role in medical therapy and disease development. Moreover, there were a total of 2696 SGBs containing a β-glucuronidase enzyme that can metabolite anti-cancer drug Gemcitabine (2′,2′-difluorodeoxycytidine) into its inactive form [47]. Besides, 456 SGBs had an agmatine gene for anti-type II diabetes drug Metformin; 225 SGBs had a tyrosine decarboxylase encoding gene for anti-parkinson drug L-dopa; and 1733 oral SGBs contained genes that can produce small molecules taurine and 5-aminovalerate which are potential drugs for autism spectrum disorder. Only a few SGBs contained *CutC*/*CutD* genes which encode key enzymes for trimethylamine, a metabolite with a high cardiovascular event risk.

### A strongly male-enriched ***Campylobacter*** species

**Figure 6:**
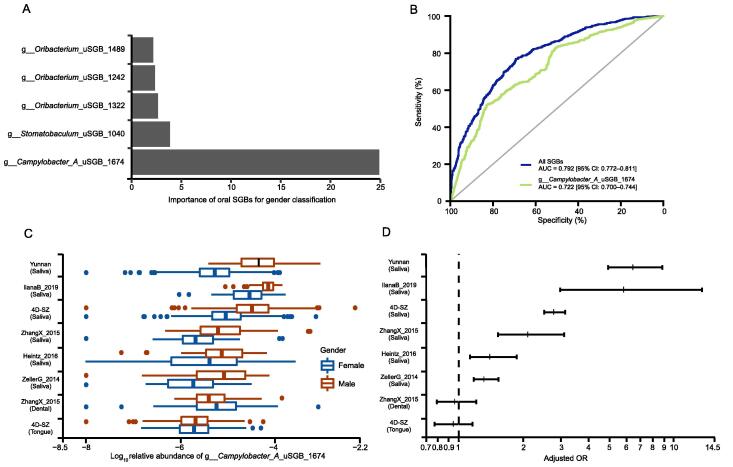
**The strongly male-enriched g__*Campylobacter_A*_uSGB_1674** **A.** The random forest importance of gender classifier. Here shows the top 5 important oral SGBs for gender classifier. **B.** AUC plot of random forest classifiers with all oral SGBs and a single uSGB g__*Campylobacter_A*_uSGB_1674. The out of bag prediction from random forest and true measure is used. **C.** Relative abundance (log_10_ transformation) of g__*Campylobacter_A*_uSGB_1674 between females and males across different studies. **D.** OR (OR > 1 means male-enriched) of g__*Campylobacter_A*_uSGB_1674 across different studies*.* g__*Campylobacter_A*_uSGB_1674 (log_10_ transformation) was regressed against gender and adjusted potential confounders such as age, BMI, and healthy status if available using generalized linear model. The middle of the bar is OR. The upper and lower limits are the 95% CI of the OR. AUC, area under curve; OR, odds ratio; CI, confidence interval; BMI, body mass index.

Gender-associated differences were observed in the saliva biochemical parameters, oral disease, and microbiota [48], [49], [50]. It is necessary to know the differences in oral microbe distribution between females and males in the natural population. We thus built a random forest classifier for gender with all 3589 oral SGBs in saliva samples of our 4D-SZ cohort (Shenzhen, China, 1025 females and 959 males). The Area Under Curve (AUC) of all SGBs for gender was 0.792 [95% confidence interval (CI): 0.772–0.811]. Four of the top 5 most important oral SGBs were from uSGBs. The random forest importance of the first ranking uSGB (g__*Campylobacter_A*_uSGB_1674) was five times more than the second ranking uSGB (g__*Stomatobaculum*_uSGB_1040) (Figure 6A)*.* The AUC of g__*Campylobacter_A*_uSGB_1674 was 0.722 (95% CI: 0.700–0.744), showing the similar predict power for gender as all 3589 oral SGBs (Figure 6B).

We next examined whether g__*Campylobacter_A*_uSGB_1674 is a conservative gender-related bacterium in saliva by integrating other existing cohort data across different populations (see Materials and methods). As shown in Figure 6C, g__*Campylobacter_A*_uSGB_1674 was detected in oral samples from multiple regions and displayed a strongly male-enriched pattern. Moreover, the difference between genders of this uSGB was only observed in saliva, but not in dental or tongue (Figure 6D). An odds ratio (OR) is a measure of association between an exposure and an outcome, and OR can be adjusted by available confounders [such as age, body mass index (BMI), and healthy status] in all cohort data by logistic regression. In our 4D-SZ cohort, the OR of g__*Campylobacter_A*_uSGB_1674 was 2.796 (OR > 1 means male-enriched; 95% CI: 2.504–3.137); the ORs of g__*Campylobacter_A*_uSGB_1674 for ZellerG_2014 (Germany and France, 93 females and 128 males) was 1.322 (95% CI: 1.175–1.531), for Heintz_2016 (Luxembourg, 47 females and 36 males) was 1.407 (95% CI: 1.131–1.865), for IlanaB_2019 (Fiji, 50 females and 52 males) was 5.913 (95% CI: 2.972–13.610), for ZhangX_2015 (Beijing, China, 121 females and 32 males) was 2.115 (95% CI: 1.524–3.099), and for Yunnan (Yunnan, China, 441 females and 229 males) was 6.533 (95% CI: 4.962–8.884). GoltsmanDSA_2018 was excluded because it only had female samples. Virulence factor (VF) analysis showed that 15 genes of 22 identified VFs in this uSGB were flagella associated, suggesting its migration ability adapted to saliva (Table S7).

We suspected that g__*Stomatobaculum*_uSGB_1674 could adapt to male-enriched metabolites. Therefore, the gene function prediction was performed based on genome annotation. As a result, 47 previously reported male-enriched metabolites [50] were involved in metabolic steps that could be catalyzed by g__*Stomatobaculum*_uSGB_1674 (Table S7).

We wondered whether g__*Campylobacter_A*_uSGB_1674 is linked to human diseases. The OR for dental calculus (1849 healthy and 429 dental calculus samples) was 1.294 (OR > 1 means disease-enriched; 95% CI: 1.163–1.443), which suggests that g__*Campylobacter_A*_uSGB_1674 in saliva is a risk of dental calculus. In contrast, the OR for type I diabetes (40 healthy and 40 type I diabetes samples) was 0.795 (95% CI: 0.649–0.938), for RA dental (dental samples collected from 51 controls and 91 RA patients) was 0.566 (95% CI: 0.408–0.756), and for RA saliva (saliva samples collected from 80 controls and 73 RA patients) was 0.598 (95% CI: 0.419–0.815) (Figure S6).

### New disease markers according to the oral genomes

To illustrate the utility of our genome collection in metagenomic studies including MWAS, we reanalyzed the dental and saliva microbiome data from RA patients and controls [8]. For better confidence in the markers regardless of the cohorts, we only analyzed the SGBs containing > 10 genomes. Similar to the original study, oral markers selected by a 5× 10-fold cross-validated gradient boosting model (GBM) included a number of Gram-negative bacteria, *e.g*., *Haemophilus* spp. and *Aggregatibacter* spp., enriched in dental samples from healthy volunteers, while only a *Pseudomonas* SGB and an *Enterococcus* SGB were selected for RA samples (Figure S7A). Interestingly, the two new RA dental markers appeared more abundant in control saliva samples. The strongest marker from healthy saliva remained *Lactococcus lactis* [7]. *Lactobacillus paracasei* and *Streptococcus infantarius* were identified, reminiscent of the beneficial effects of *Lactobacillus casei* gavage in a rat model of RA [51], [52]. The assembled genomes allowed matching of different species in the *Veillonella* genus as RA saliva markers. Moreover, *Pauljensenia*, a genus recently renamed from *Actinomyces* [53], was identified as a highly predictive marker of RA. As *Actinomyces* spp. are the basis for dental attachment of oral bacteria [54], the potential contribution of *Pauljensenia* spp. to periodontitis in RA patients remains to be explored. In addition, the dental microbiome was obviously deranged, consistent with epidemiology [7].

A set of saliva samples from CRC patients and controls from France are also available [55]. Here, we found *Pauljensenia* spp. to be control-enriched, along with *Acinetobacter radioresistens*, *Lachnoanaerobaculum* sp., and *Catonella* sp., *etc*. (Figure S7B). *Streptococcus thermophilus*, a species previously found to be enriched in fecal samples from control or adenoma compared to CRC patients [56], was also identified in control saliva. The markers enriched in CRC oral samples were more unexpected than CRC fecal samples. Besides *Porphyromonas* spp. and *Prevotella maculosa*, we found a *Lachnospiraceae* SGB (potentially TMA-producing and consistent with gut results [10], [57], [58], [59]), *Capnocytophaga leadbetteri*, and *Cardiobacterium hominis*, *etc*. (Figure S7B). Thus, the substantially expanded collection of oral microbial genomes enabled the discovery of new disease markers and the genomic representation of previously reported markers, facilitating the shift from fecal to oral microbiome-based diagnosis and therapeutics.

## Discussion

In summary, we provided the largest set of oral metagenomic shotgun data, assembled 56,213 draft genomes for the human oral microbiome, including 2313 new species as well as many new strains of known species. More than 94.36% of metagenomic reads can now be mapped to the expanded genome catalog for oral microbiomes, enabling much more comprehensive profiling of these communities. Many uSGBs are not currently represented by cultured isolates. The results illustrate that culturomics has not even exhausted the microbial complexity in the more accessible body sites and that metagenomic data for large cohorts of non-fecal samples have great potential. A number of taxa with compact genomes were identified in this study, such as CPR and Mollicutes. Much remains to be elucidated for the metabolic requirement of small bacteria in the oral microbiome. Oral bacteria also contribute to the discovery of new CRISPR-Cas systems [60]. Species with thousands of metagenomic and isolated genomes would be amenable to microbial genome-wide association studies [61] to discover virulence factors, drug resistance, and more commensal functions, which has so far only be possible for pathogens.

In this study, we identified a conservative saliva- and male-specific uSGB g__*Campylobacter_A*_uSGB_1674 across cities and countries. It was assembled from our 4D-SZ cohort. What is more interesting is that g__*Campylobacter_A*_uSGB_1674 is only significantly different in saliva, not in tongue or dental. Genome analysis showed that this saliva-specific difference of gender may be due to its flagella enrichment function which powers its swimming ability in saliva. Function annotation by eggNOG showed that many male-enriched metabolites can be catalyzed by g__*Campylobacter_A*_uSGB_1674, suggeting that it can be more adaptive to male oral metabolome. Further experimental validation of this microbe should be carried out to verify its potential functions.

Although we have reconstructed tremendous MAGs, the oral metagenomic assembly still faces many challenges. For example, the host rate is about 80% in saliva, and high-quality assembly requires further expanded sample size and sequencing depth (Figure S2B). Many homos read contamination were still found after host removal in public data (Figure S3A). A more sensitive host removal method is needed to reduce the influence of host sequences. The oral cavity contains diverse forms of microbes such as protozoa and fungi (like *Candida* as a common oral infection in the immune deficiency population and infants). However, no fungus was observed from our data, probably due to a lack of such samples. Our new oral genome set enables us to identify new biomarkers for RA and CRC, but further validation would depend on more patient samples. By collecting more samples with a variety of diseases, the diversity and quality of the oral genome set will be further improved and would benefit human society by providing health management and disease prevention.

## Materials and methods

### Collection of the oral microbiomic samples

The 2675 oral metagenomic samples (2284 saliva and 391 tongue samples) from the Chinese 4D-SZ cohort and 671 saliva samples from the Yunnan cohort were newly collected in this study from 2017 to 2018 (Table S1, sheets 4 and 5). The 4D-SZ cohort is a young cohort composed of 2675 people from Shenzhen, China. In those people, 2162 people have age and gender information, with an average age of 30.27 (± 5.58) years old, including 1051 males and 1111 females. No complex diseases were observed in this cohort. And the population of the Yunnan cohort comes from six regions in Yunnan Province of China with an average age of 31.75 (± 10.78) years old, including 260 males and 487 females.

The 4D-SZ samples were self-collected by the volunteers during a physical examination using the MGIEasy Fecal Sample Collection Kit (Catalog No. 10000035265, BGI, Shenzhen, China) containing a room temperature stabilizing reagent to preserve the metagenome [62]. For the saliva sample, a double concentration of stabilizing reagent kit was used and 2 ml saliva was collected. The samples from Yunnan Province were self-collected using a commercial kit (Catalog No. 401103, Zeesan, Xiamen, China). Yunnan samples were approved by The First Affiliated Hospital of Kunming Medical University, China. The collected samples were temporarily stored in freezers at −80 °C and then transported to China National GeneBank (CNGB), Shenzhen on dry ice via commercial logistics.

### DNA extraction, sequencing, and quality control

DNA extraction of the stored samples within the next few months was performed using the MagPure Stool DNA KF Kit B (Catalog No. MD5115, Magen, Guangzhou, China) from 1 ml of each sample [63]. Metagenomic sequencing was done on the BGISEQ-500 platform (BGI, Shenzhen, China) [64] (100 bp of paired-end reads for all samples and four libraries were constructed for each lane) and generated 101.4 billion pairs of raw reads (mean: 75.8 million paired-end reads per sample; standard deviation: 14.1 million paired-end reads). DNA was extracted in the same way as above. Sequencing was performed on the BGISEQ-500 machines and generated 26.5 billion single-end 100 bp length reads (mean: 39.5 million single-end reads per sample; standard deviation: 8.2 million single-end reads). After filtering and trimming with strict filtration standards (not less than mean quality phred score 20 and not shorter than 51 bp read length) using fastp (v0.19.4) [65], host read contamination removing using Bowtie2 (v2.3.5) [66] (human genome GRCh38) and seqtk (v1.3) [67], and quality control, we totally got 54.9 billion high-quality paired-end reads and 7.1 billion high-quality single-end reads.

### Collection of public metagenomic samples

A total of 808 public oral metagenomic datasets were downloaded from NCBI Sequence Read Archive database (SRA: SRP029441, ERP006678, SRP133047, ERP110622, and SRP07256), which came from five different studies [8], [21], [22], [23], [24] (Table S1, sheet 3) that have been reported previously. These published data were used to do assembly and profile.

To illustrate the representativeness of the assembled genome set for new data, additional 81 oral metagenomes from three validation cohorts were downloaded from NCBI SRA database (SRA: ERP016024, SRP018108, and SRP052958) (Table S1).

### Metagenomic ***de novo*** assembly, binning, and quality assessment

The high-quality paried-end and single-end reads were individually assembled using the assembly module of metapi pipeline with different max kmer cutoff by different max read length of each sample applying SPAdes (v3.13.0) [68] (paired-end reads with option --meta [25]). After we obtained draft genomes on the contig level of each sample, the reads were mapped back to each assemblies using BWA-MEM (v0.7.17) [69] with default parameters and the contig depth was calculated by jgi_summarize_bam_contig_depths. Then using MetaBAT2 (v2.12.1) [26], we performed metagenomic binning individually for each sample. Finally, we obtained a total of 163,718 bins. After MAG quality assignment by CheckM (v1.0.12) [28] lineage workflow, 15,013 high-quality (completeness > 90% and contamination < 5%) bins and 41,200 medium-quality (completeness > 50% and contamination < 10%) bins (Table S2) were generated based on MIMAG standard [27]. The 16S rRNA sequences in the MAGs were searched by Barrnap (v0.9; https://github.com/tseemann/barrnap) with parameters “--reject 0.01 --evalue 1e-3”, and the tRNA sequences in the MAGs were searched by tRNAscan-SE (v2.0.3) [70] with the default parameters.

### Clustering metagenomic genomes into SGBs

The 56,213 reconstructed genomes and 190,309 reference genomes were grouped into SGBs by a two-step clustering strategy as reported previously [18] with a slight modification. In the first step, an all-versus-all genetic distance matrix between the 246,522 genomes was carried out using Mash (v2.0) [71] (“-k 21 -s 1e4” for sketching). Then, hierarchical clustering with average linkage and genetic distance cutoff of 0.05 on the distance matrix by fastcluster [72] generated 33,008 clusters. Because the Mash will underestimate the distance between the incomplete genomes [73] and split same-species genomes into multiple SGBs, we performed clustering base on ANI in the second step. First, we divided the SGBs into kSGBs and uSGBs according to with or without reference genomes. Then, a representative genome was selected for each SGB. For the kSGB, the genome with the largest genome size was selected. For the uSGB, all MAGs were ranked by completeness (in descending order), contamination (in ascending order), coverage (in descending order), strain heterogeneity (in ascending order), and N50 (in descending order), and a representative genome was selected as the one minimizing the sum of the five ranks. We recalculated the more precise genetic distance using pyani (v0.2.9) [74] (option ‘-m ANIb') for the pairs of representative genomes with mash distances less than 0.95 and only left ANI with genome coverage above 0.3. Following hierarchical clustering with complete linkage based on ANI > 95%, 12,911 representative genomes with mash distances less than 0.95 were merged to 11,427 new clusters. Finally, we obtained 31,525 SGBs by a two-step clustering strategy. In this dataset, only 3589 SGBs included eHOMD genomes or oral MAGs, and they were named as oral SGBs and further divided into 2313 uSGBs and 1276 kSGBs.

### Reconstruction of the human oral microbiome phylogenetic structure

The phylogenetic trees of 3589 representative genomes of SGBs (Figure 3A) and 76 genomes of the Acholeplasmataceae family were both built using the 400 PhyloPhlAn markers with the parameters “--diversity high --fast --min_num_markers 80” by the PhyloPhlAn2 [75]. As input data for PhyloPhlAn2, proteomes were predicted using Prodigal (v2.6.3) [37] with default parameters. Following tools with their set of parameters were used in the configuration files: Diamond (v0.9.22.123) [76] with the parameters “blastp --quiet --threads 1 --outfmt 6 --more-sensitive --id 50 --max-hsps 35 -k 0”; Mafft (v7.407) [77] with the “--anysymbol” option; Trimal (v1.4.rev15) [78] with the “-gappyout” option; Iqtree (v1.6.12) [79] with the parameters “-quiet -nt AUTO -m LG”. After that, 3433 representative genomes of SGBs contained more than 80 markers, and the phylogenetic tree in Figure 3A was generated using GraPhlAn (v1.1.3) [80]. All genomes of the Acholeplasmataceae family were remained and the phylogenetic tree in Figure 4A was generated using FigTree (v1.4.4).

### Taxonomic and function analyses of SGBs

The taxonomic classification of 3589 representative genomes of SGBs was assigned using the GTDB-Tk (v0.3.2) [36], [37], [38], [39], [40] classification workflow with external GTDB release 89. Although some kSGBs already have taxonomy labels, we still used GTDB-Tk to classify them, because GTDB-Tk has its own taxonomy classification system that is different from the NCBI taxonomy database. Then above the genus level, we manually removed the classification tag with a single letter suffix (Table S3). Those suffixes were used to indicate the taxa needed to be subdivided based on the current GTDB reference tree. We used eggNOG mapper (v1.0.3) [42] to do genome-wide functional annotation through orthology assignment.

### Mapping rate comparison between different oral-related genome databases

The mapping rates of oral metagenomic reads align to four different oral-related genome databases (eHOMD, SGBs from Pasolli et al. [18], oral SGBs, and oral genome catalog) were compared based on the statistics summary of Bowtie2's results (Table S4). First, we randomly selected 100 oral metagenomic samples from each of the 4D_SZ and Yunnan cohorts. With all 808 public samples and 81 additional verified samples which haven’t been assembled (Table S1, sheet 2), a total of 1089 oral metagenomic samples were mapped to these databases respectively using Bowtie2 (v2.3.5) with SE model (-U) and default parameters. For acceleration calculation, all 142,723 oral genomes were divided into 19 subsets and indexed by Bowtie2. Reads were aligned to these databases in turns, and only unmapped reads (samtools fastq -f 4) were retained for the next alignment.

### Taxonomic assignment for unmapped reads by Kraken2

Kraken2 is a taxonomic classification system using exact *k*-mer matches and has a higher error tolerance in alignment than Bowtie2. The reads which were unmapped to oral genome set were aligned to the database (maxikraken2_1903_140GB, March 2019) which includes archaea, bacteria, fungi, protozoa, viral, and human from Loman Lab (https://lomanlab.github.io/mockcommunity/mc_databases.html) by Kraken 2.0.8-beta with default parameters.

### Metapi for oral SGB metagenomic profiling

The quantification of species relative abundance of oral metagenomic samples was performed with the taxonomic profiling module of metapi pipeline: 1) build the oral genome index of oral representative SGBs by Bowite2; 2) align the high-quality reads of each sample to the oral genome index using Bowtie2 with the parameters “--end-to-end --very-sensitive --seed 0 --time -k 2 --no-unal --no-discordant -X 1200”; 3) obtain the normalized contig depths by using jgi_summarize_bam_contig_depths; and 4) convert the normalized contig depth to the relative abundance of each SGB for each sample base on the correspondence of contigs and genome. Finally, we merged the relative abundances of all representative SGBs to generate a taxonomic profile.

### Principal coordinate analysis and heatmap for human oral profile

Principal coordinate analysis (PCoA) of human oral profile was done using the dudi.pco function in ade4 R package based on bray distance from vegan 2.5.2 R package. The mean top 10 most abundant SGBs from every study were merged (total 27 SGBs) to visual in the pheatmap R package.

### Pangenome and phylogenetic analyses of kSGBs and uSGBs

From the taxonomic profiling results of 4154 oral meta-genomic samples, the most prevalent eight genera were selected based on the ranking of average relative abundance (in descending order), occurrence frequency (in descending order), and oral genome number/SGB size (in descending order), including *Prevotella*, *Neisseria*, *Streptococcus*, *Veillonella*, *Porphyromonas*, *Fusobacterium*, *Pauljensenia*, and *Haemophilus*. Then, we chose the top 10 prevalent species for each genus to do pangenome analysis. All genomes of each SGB were annotated by prokka (v1.13.7) [81] and constructed to the pangenome database via PanPhlAn (v1.2) [82]. Finally, the gene-family presence/absence profile matrix was transformed to a zero/one matrix for reference genomes and the genomes of each SGB were reconstructed to perform rarefaction analysis. Accumulation curves (Figure S5) based on the number of core genes of each SGB were bootstrapped ten times at each sampling interval. The observation of the intra-SGB phylogenetic structure of *Neisseria sp000186165*_kSGB_3225, *Prevotella nanceiensis*_kSGB_3467, and *Porphyromonas* unclassified_kSGB_3273 was performed by the nonmetric multidimensional scaling analysis using the metaMDS function of R package vegan (v2.5.2).

### Identification of a novel saliva- and male-specific oral uSGB g__***Campylobacter_A***_uSGB_1674

We built a RandomForest classifier for gender with 3589 oral SGBs in saliva samples of our 4D-SZ cohort (1025 females and 959 males) by randomForest 4.6-14 R package. Receiver operating characteristic (ROC) curve was plotted with pROC R package. The most importance SGB for the gender classifier was identified to be g__*Campylobacter_A*_uSGB_1674 (Figure 6A). Generalized linear model (GLM) analysis was performed to further confirm the association between g__*Campylobacter_A*_uSGB_1674 and gender after adjusting potential confounders. The ORs extracted from the GLM were plotted in Figure 6D with R package ggplot2. The association between g__*Campylobacter_A*_uSGB_1674 and diseases was also tested with GLM after adjusting confounders and the ORs were plotted in Figure S6. For 4D-SZ, we adjusted age, antibiotics usage during last six months, dietary_structure, disease_oral_ulcer, disease_caries, BMI, dental_calculus, inflammatory_gingivitis, and decayed_tooth. For ZellerG_2014 and Heintz_2016, we adjusted age and healthy status. For IlanaB_2019 and Yunnan, we adjusted age only. For ZhangX_2015, we adjusted age, BMI, and healthy status.

### Disease markers according to the oral genomes

The metagenome-wide association between 3589 SGB profiles and diseases for previously published CRC and RA studies was done using a GLM with adjustment for potential confounders such as gender, age, and BMI (Table S1). BMI is only available for RA. Species relative abundance was asin-sqrt transformed as previously described [83]. Non-oral SGBs were excluded. Correction for multiple hypothesis tests was done using false discovery rate (FDR). We predicted disease status using GBM in the caret R package, such that 80% of the samples were randomly sampled for each estimator. The depth of the tree at each estimator was not limited, but leaves were restricted to have at least 30 instances. We used 4000 estimators with a learning rate of 0.002. All the oral marker SGBs with FDR < 1% were included in the model as predictors. To avoid overfitting, 5× 10-fold cross-validated ROC was used to measure the model performance. VarImp function was used to extract the GBM importance.

## Ethical statement

The study was approved by the Institutional Review Board (IRB) of BGI-Shenzhen (Nos. BGI-IRB19121 and BGI-IRB17162) and the ethics committee of No.1 Affiliated People's Hospital of Kunming Medical University [(2017) Ethics review L No.14], China. Informed consent was obtained from each participant.

## Code availability

The pipeline used in this study is available at https://github.com/ohmeta/metapi. The scripts of figures are available at https://github.com/ohmeta/oral-assembly.

## Data availability

All sequence data are available at CNGB Sequence Archive (CNSA) of China National GeneBank DataBase (CNGBdb) (CNSA: CNP0000687 for the 4D-SZ cohort and CNP0001221 for the Yunnan cohort). Oral SGB genomes and genome annotations are available at Microbiome Database of National Genomics Data Center (https://cngb.org/microbiome/genomecatalog/human_oral/). Oral SGB genomes and genome annotations have also been deposited in the Genome Sequence Archive [84] at the National Genomics Data Center, Beijing Institute of Genomics, Chinese Academy of Sciences / China National Center for Bioinformation (BioProject: PRJCA003731), and are publicly accessible at https://ngdc.cncb.ac.cn/gsa.

## CRediT author statement

**Jie Zhu:** Conceptualization, Methodology, Software, Visualization, Writing - original draft. **Liu Tian:** Conceptualization, Methodology, Visualization, Writing - original draft. **Peishan Chen:** Investigation. **Mo Han:** Investigation. **Liju Song:** Investigation. **Xin Tong:** Investigation. **Xiaohuan Sun:** Investigation, Writing - review & editing. **Fangming Yang:** Investigation. **Zhipeng Lin:** Investigation. **Xing Liu:** Investigation. **Chuan Liu:** Investigation. **Xiaohan Wang:** Investigation. **Yuxiang Lin:** Investigation. **Kaiye Cai:** Investigation. **Yong Hou:** Supervision. **Xun Xu:** Supervision. **Huanming Yang:** Supervision. **Jian Wang:** Supervision. **Karsten Kristiansen:** Writing - review & editing. **Liang Xiao:** Supervision. **Tao Zhang:** Supervision. **Huijue Jia:** Conceptualization, Writing - review & editing, Supervision, Project administration. **Zhuye Jie:** Conceptualization, Methodology, Visualization, Writing - original draft, Project administration. All authors have read and approved the final manuscript.

## Competing interests

The authors have declared no competing interests.

## Supplementary data

Supplementary Figure S1 **Complete set of rRNAs and tRNAs in MAGs A.** The number of standard 20 amino acids are decoded by tRNAs across the MAGs. **B.** Three types of rRNA were predicted across the MAGs.

Supplementary Figure S2 **Summary of assembly quality and SGBs distribution across 7 studies A.** The uSGBs origin distribution across studies. The number of uSGBs from each study are shown in brackets. **B.** Correlation between the number of high-quality and medium-quality MAGs and the sequence bases after host removal. Each point represents a sample and is colored by studies. The shape of point represents the oral site of sample. Regression curve was generated from all samples and the shaded regions representing the 95% confidence level intervals. **C.–E.** The number of SGBs (> 0.1% abundance), uSGB richness (number of uSGB/number of all SGB), sum of uSGB abundance per sample for each study.

Supplementary Figure S3 **The unmapped reads classification A.** The ratios of unmapped reads were classified as *homo sapiens* by Kraken2. **B.** Pie chart of Kraken2 taxonomic classification of unmapped reads from Fiji. Genera in Fiji samples which Kraken2 relative abundance ≥ 0.1% is showed in bar chart.

Supplementary Figure S4 **The COG functional annotation from all MAGs in the new candidatus family** Candidatus Bgiplasma function genome was annotated by EggNOG mapper. The function categories of COG are displayed as the percentage of genes annotated to that category. COG, clusters of orthologous genes.

Supplementary Figure S5 **The pangenome genetic diversity** The gene number rarefaction curve for SGBs in the eight most prevalent genus. X-axis is the ratio of randomly selected genomes divide total genome numbers in each SGB. Solid lines are only included public reference genomes in SGBs. Dashed lines are included both public reference genome and our reconstructed MAGs in SGBs. Curves are colored by SGBs.

Supplementary Figure S6 **MWAS analysis between the strongly male-enriched g__*Campylobacter_A*_uSGB_1674 and human disease** OR (OR > 1 means disease-enriched) of g_*_Campylobacter_A*_uSGB_1674 across different studies. g__*Campylobacter_A*_uSGB_1674 (log_10_ transformation) was regressed against disease statue and adjusted potential confounders such as age, BMI, and gender if available using generalized linear model. The middle of the bar is OR. The upper and lower limits are the 95% CI of the OR. MWAS, Metagenome-wide association studies.

Supplementary Figure S7 **Disease markers according to the oral genomes A.** SGBs association with RA. Left panel, the Manhattan plot shows metagenomic wise association of oral SGBs for RA. The species are ordered according to their phylogeny (bottom) and the association direction (left side of the specie name are negative and right side of specie name are positive). Each point is one SGB and point height indicates the FDR value correction for multiple hypothesis tests from a generalized linear model test between diseased and healthy species abundance after adjusting age, gender, and BMI. Right panel, SGBs association with RA. We select 40 large (> 10 genomes) and most importance oral SGBs for disease prediction using GBM. The species are order according to their partial spearman correlation adjusted age, gender, BMI, and GBM importance. The bar length indicated the FDR value between groups as described above. The dotted line indicates the FDR of 1%. The red square in bar is the sqrt GBM importance and share the same X-axis with FDR value. uSGBs are highlight in bold label text. **B.** SGBs association with CRC. FDR, false discovery rate. GBM, gradient boosting machine.

Supplementary Table S1 **Sequence data or meta data of samples used in this study**

Supplementary Table S2 **Summary of assembly and quality for 56,213 MAGs over medium-quality**

Supplementary Table S3 **SGBs cluster, structure, and taxonomic assignment**

Supplementary Table S4 **Mappability of oral metagenomes between different databases**

Supplementary Table S5 **The genomics characteristics of the new candidatus family**

Supplementary Table S6 **Genes in oral genome association with human drugs and disease and SGB-drug profile**

Supplementary Table S7 **Metabolite and virulence factor annotation of g__Campylobacter_A_uSGB_1674**
