# Supplementary figures and images for: Over 50,000 Metagenomically Assembled Draft Genomes for the Human Oral Microbiome Reveal New Taxa
Source: Genomics Proteomics Bioinformatics. 2021 Sep 4;20(2):246–59. doi: 10.1016/j.gpb.2021.05.001 (PMC9684161; doi:10.1016/j.gpb.2021.05.001)

A

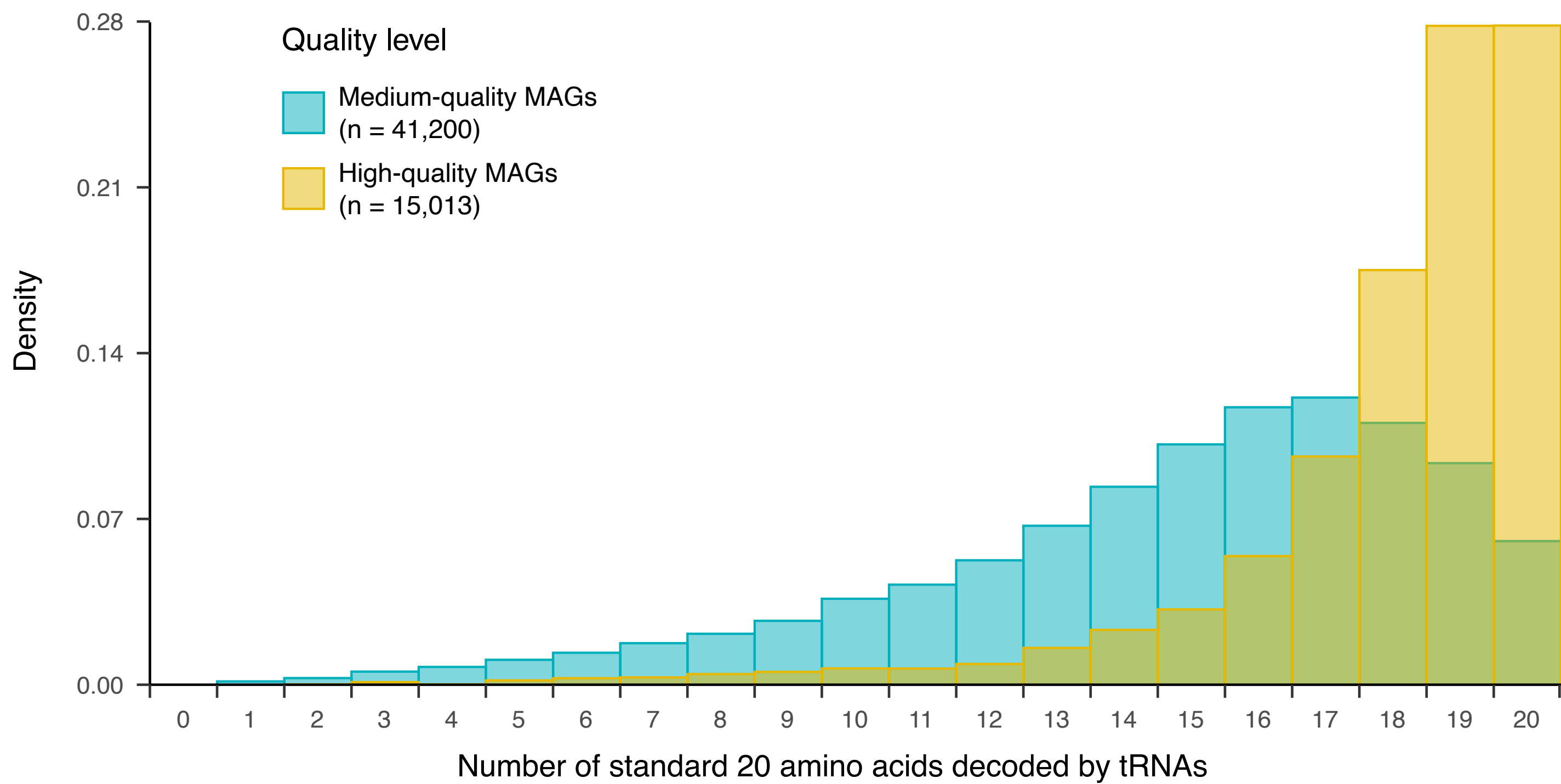

B

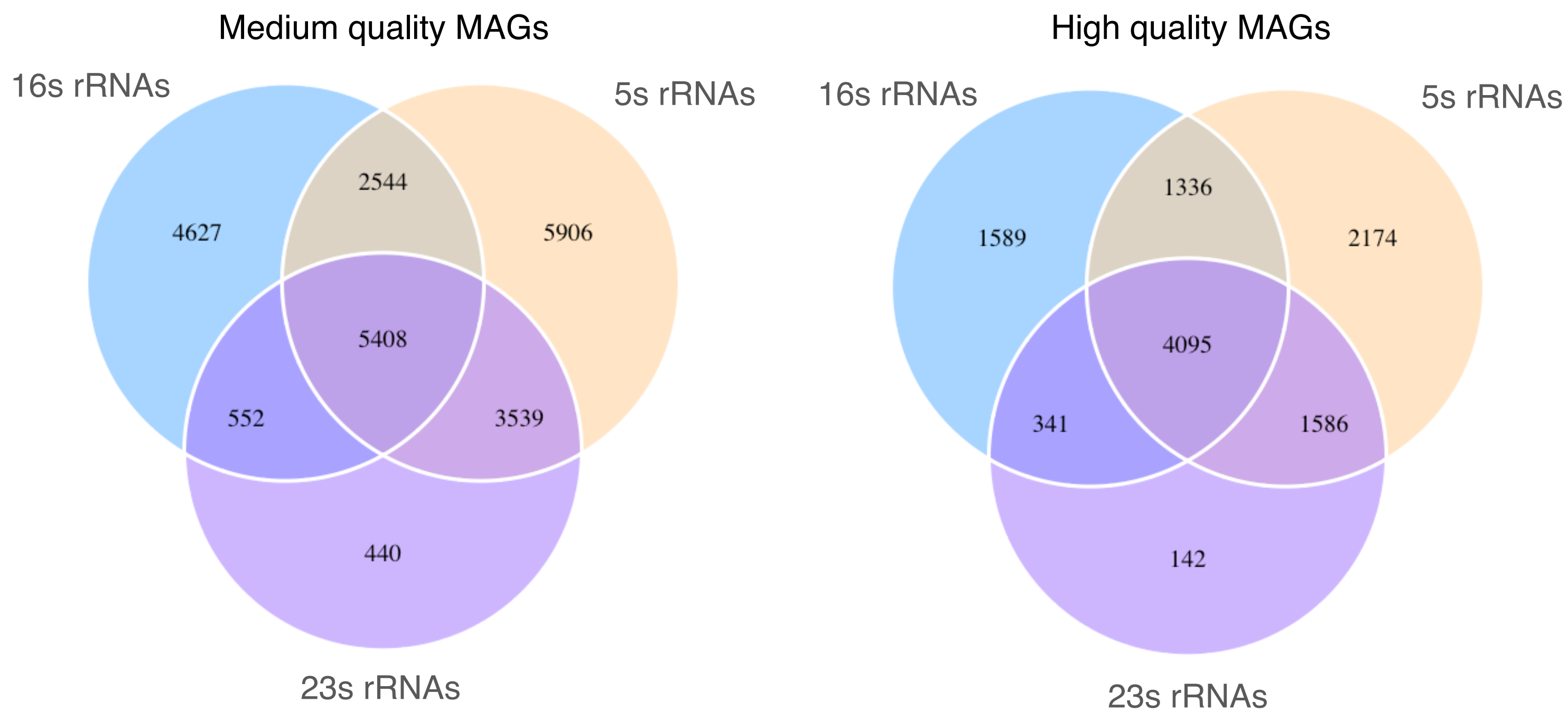

Supplement: Supplementary Figure S1 — Complete set of rRNAs and tRNAs in MAGs A. The number of standard 20 amino acids are decoded by tRNAs across the MAGs. B. Three types of rRNA were predicted across the MAGs. [file mmc1.pdf]

**A**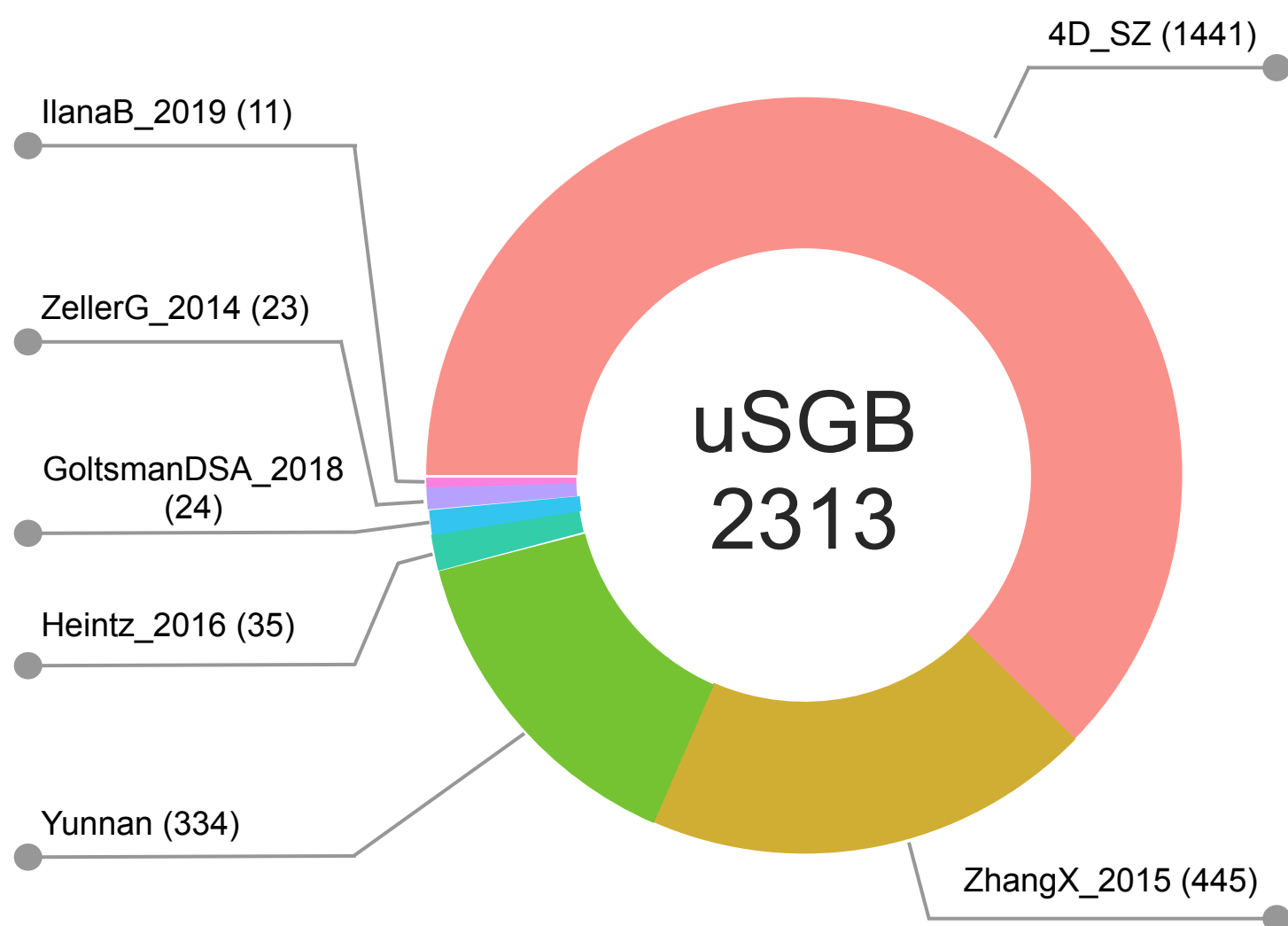**B**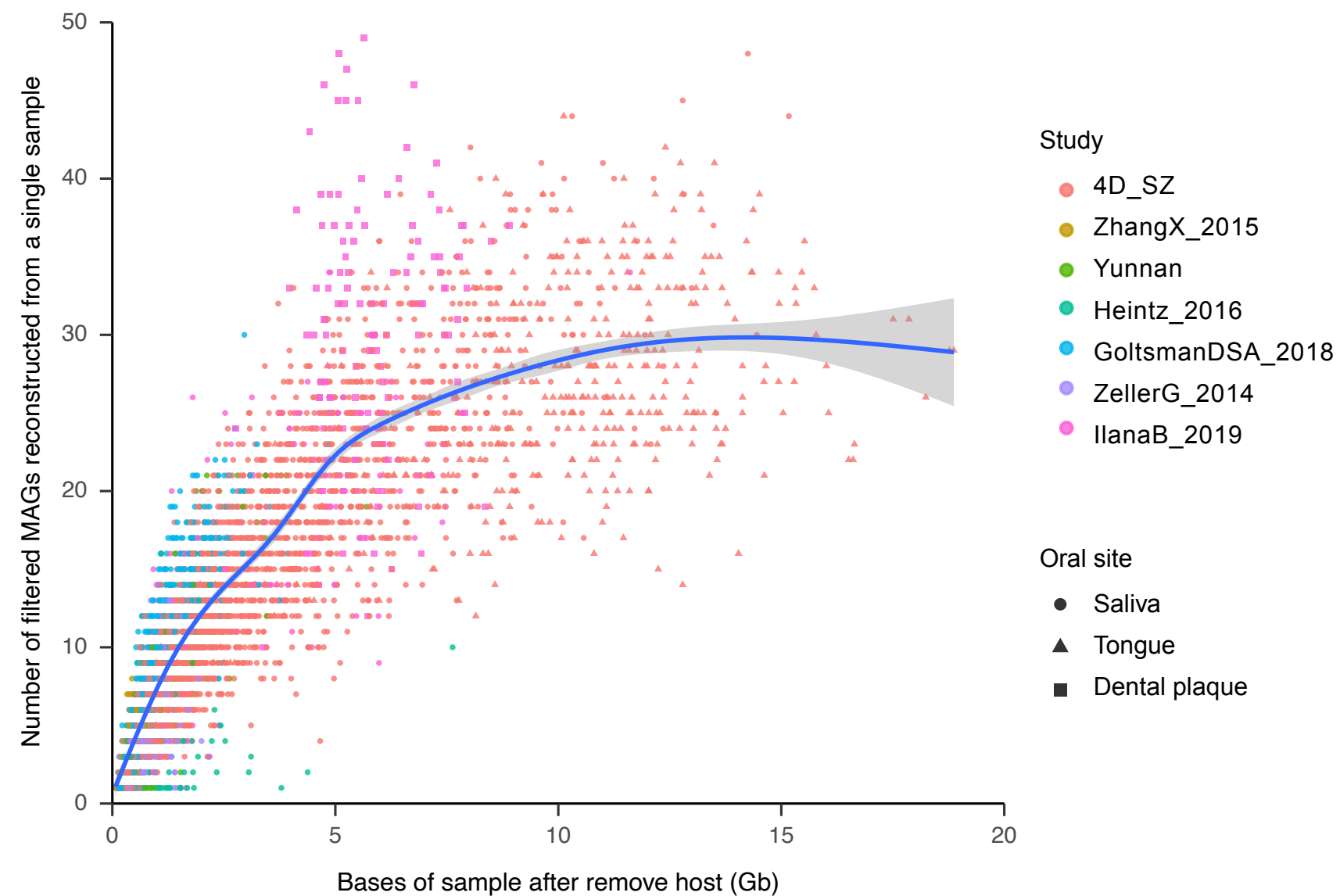**C**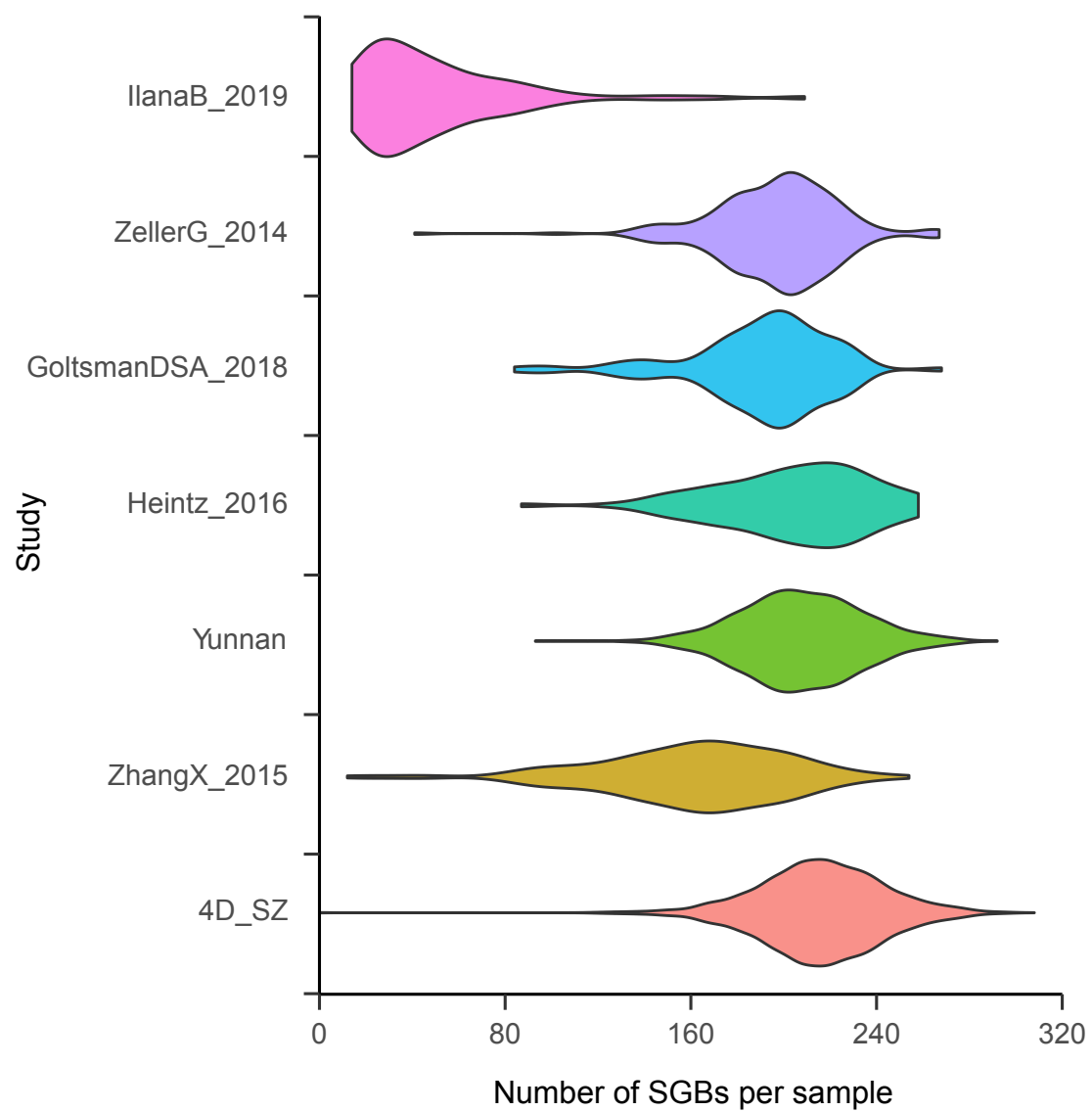**D**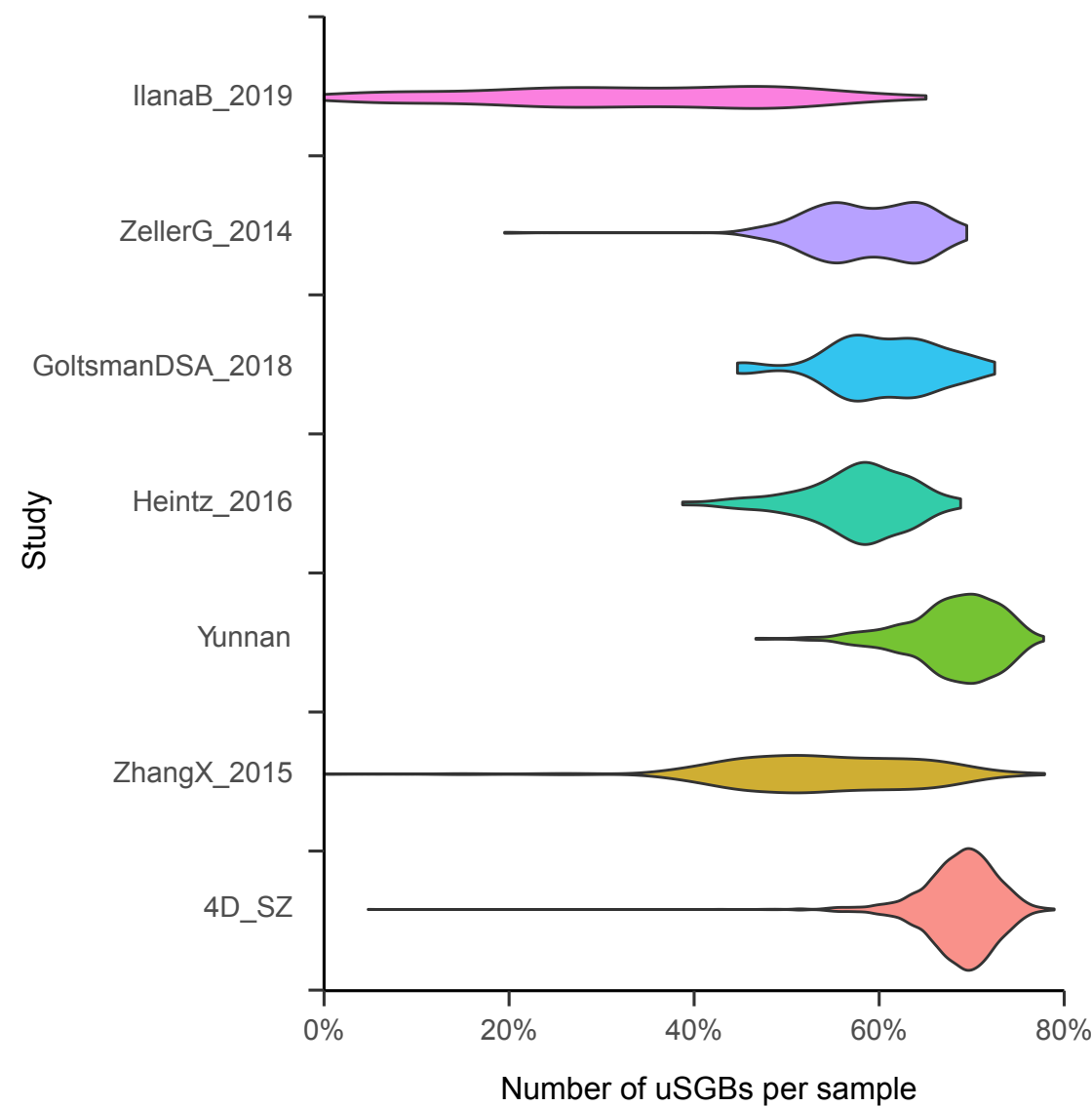**E**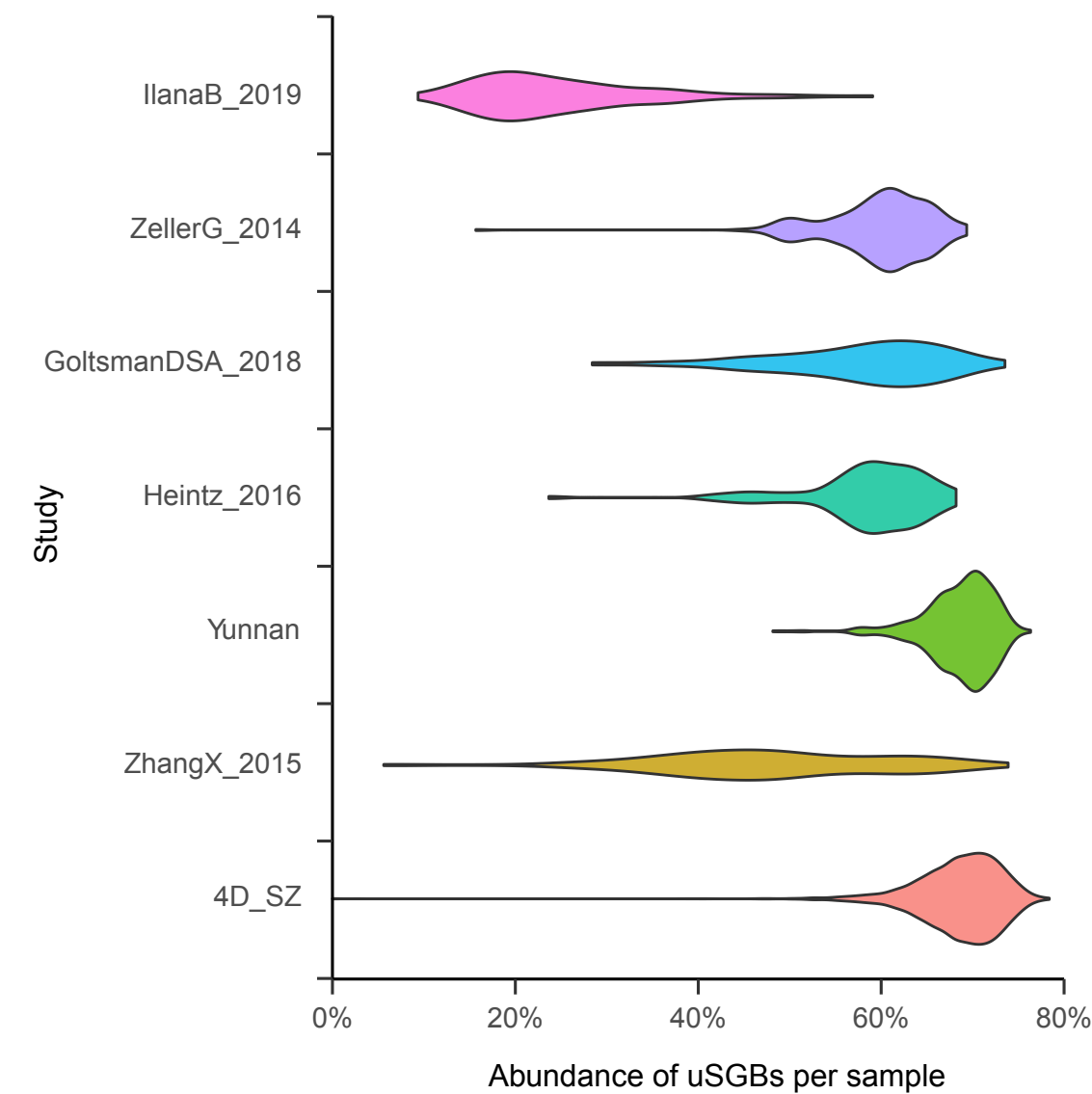

Supplement: Supplementary Figure S2 — Summary of assembly quality and SGBs distribution across 7 studies A. The uSGBs origin distribution across studies. The number of uSGBs from each study are shown in brackets. B. Correlation between the number of high-quality and medium-quality MAGs and the sequence bases after host removal. Each point represents a sample and is colored by studies. The shape of point represents the oral site of sample. Regression curve was generated from all samples and the shaded regions representing the 95% confidence level intervals. C.–E. The number of SGBs (> 0.1% abundance), uSGB richness (number of uSGB/number of all SGB), sum of uSGB abundance per sample for each study. [file mmc2.pdf]

A

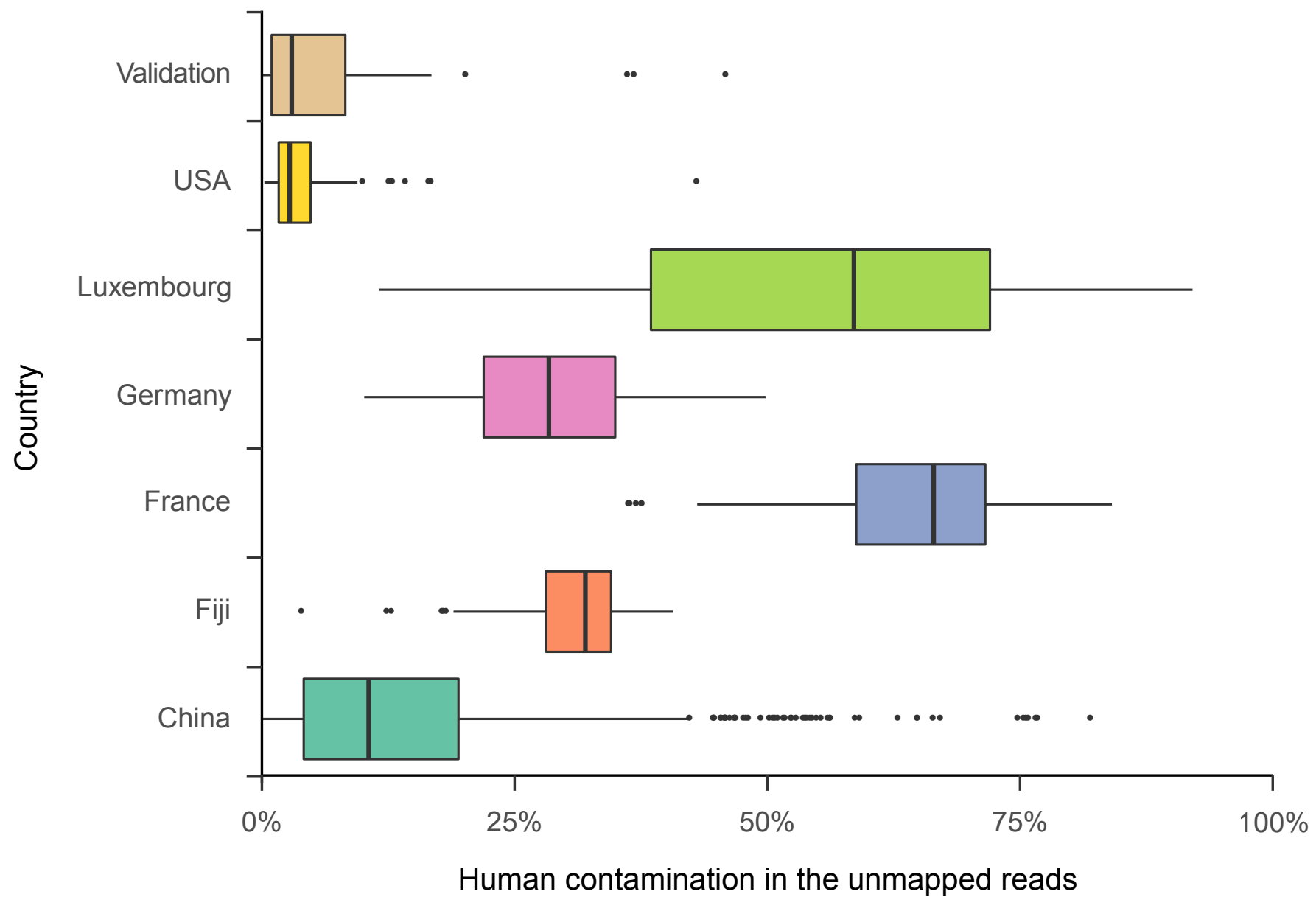

B

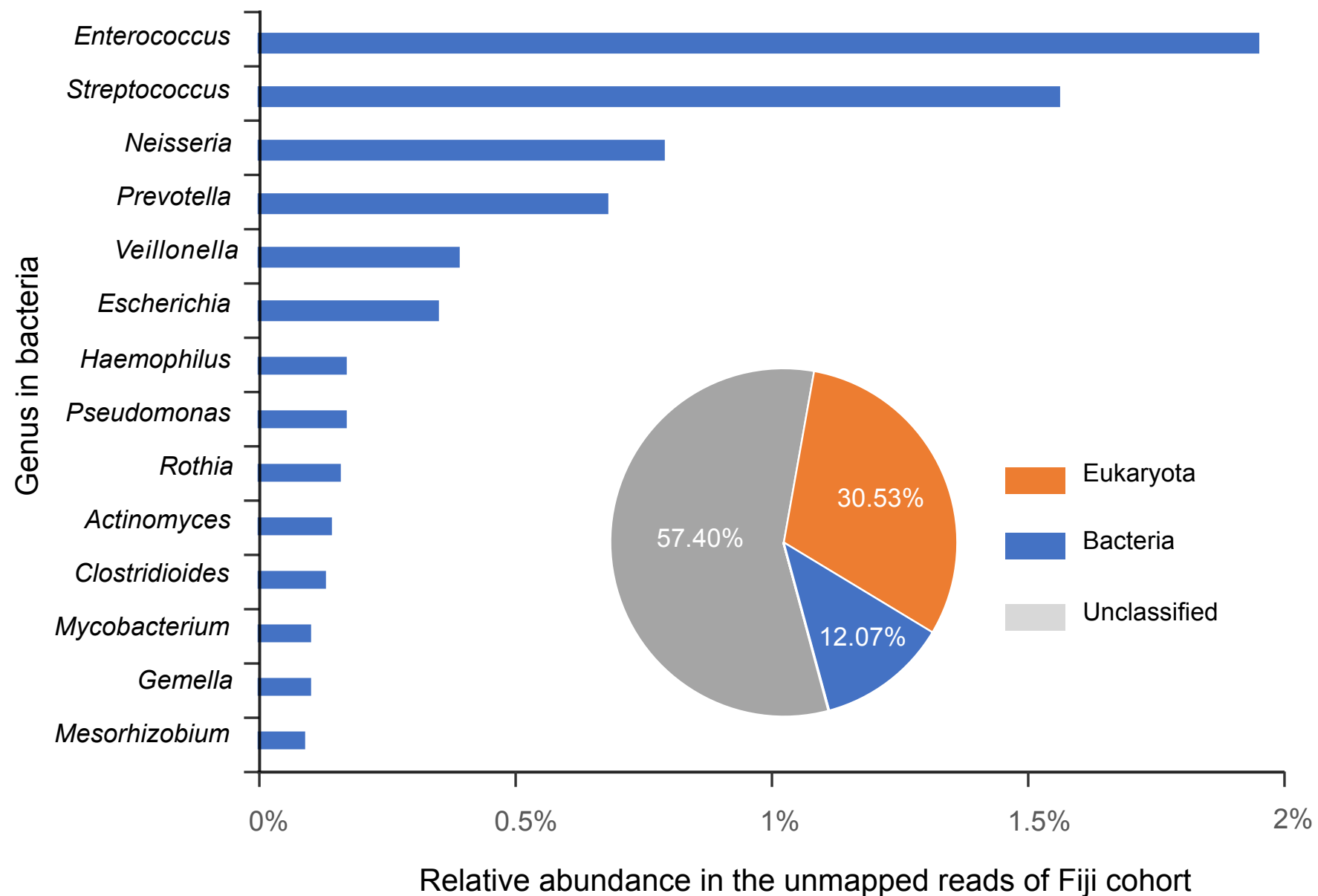

Supplement: Supplementary Figure S3 — The unmapped reads classification A. The ratios of unmapped reads were classified as homo sapiens by Kraken2. B. Pie chart of Kraken2 taxonomic classification of unmapped reads from Fiji. Genera in Fiji samples which Kraken2 relative abundance ≥ 0.1% is showed in bar chart. [file mmc3.pdf]

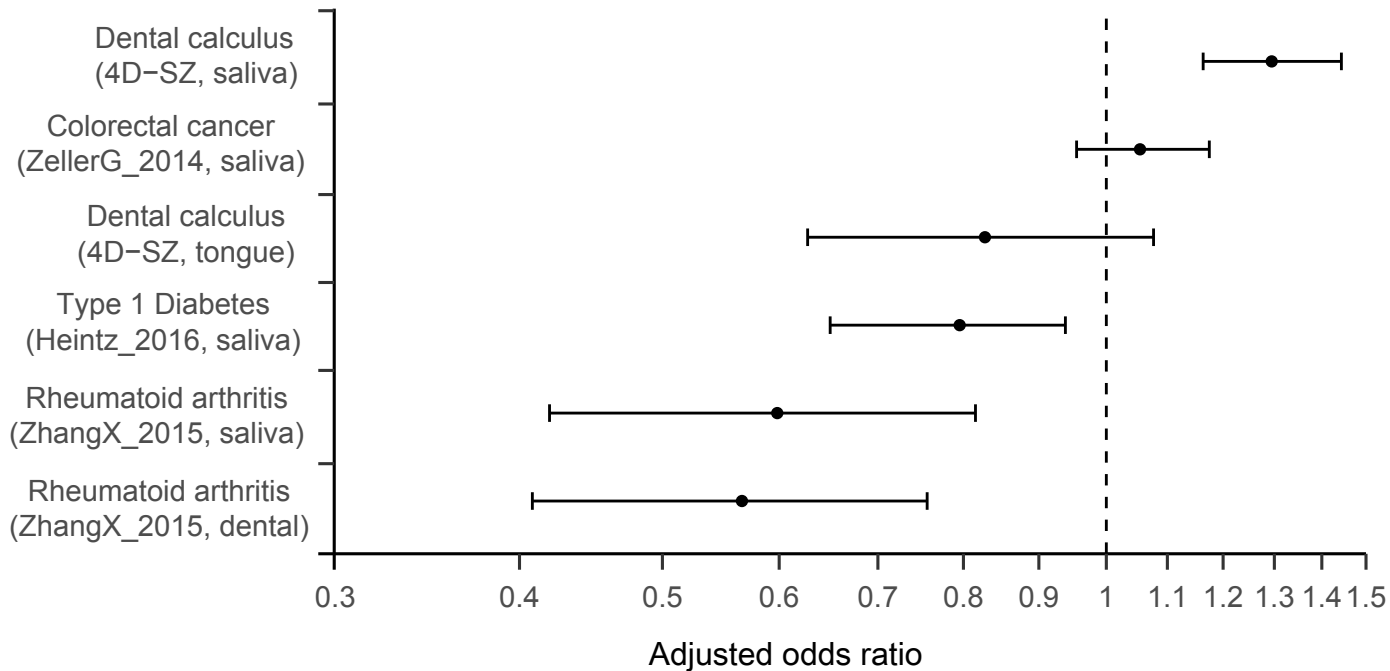

Supplement: Supplementary Figure S6 — MWAS analysis between the strongly male-enriched g__Campylobacter_A_uSGB_1674 and human disease OR (OR > 1 means disease-enriched) of g__Campylobacter_A_uSGB_1674 across different studies. g__Campylobacter_A_uSGB_1674 (log10 transformation) was regressed against disease statue and adjusted potential confounders such as age, BMI, and gender if available using generalized linear model. The middle of the bar is OR. The upper and lower limits are the 95% CI of the OR. MWAS, Metagenome-wide association studies. [file mmc6.pdf]
