# Supplementary material for: Over 50,000 Metagenomically Assembled Draft Genomes for the Human Oral Microbiome Reveal New Taxa
Source: Genomics Proteomics Bioinformatics. 2021 Sep 4;20(2):246–59. doi: 10.1016/j.gpb.2021.05.001 (PMC9684161; doi:10.1016/j.gpb.2021.05.001)
Supplement: Supplementary Figure S4 — The COG functional annotation from all MAGs in the new candidatus family Candidatus Bgiplasma function genome was annotated by EggNOG mapper. The function categories of COG are displayed as the percentage of genes annotated to that category. COG, clusters of orthologous genes. [file mmc4.pdf]

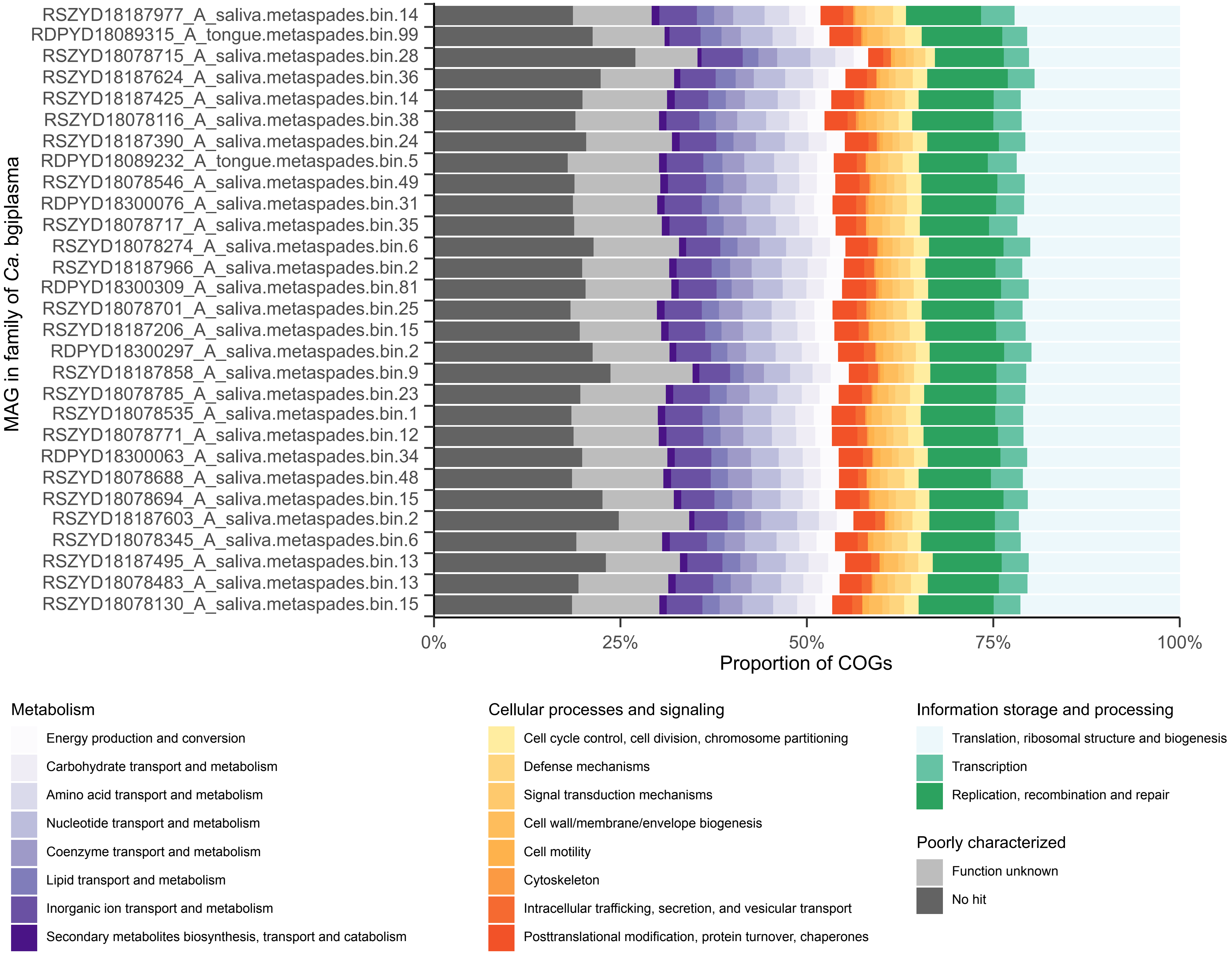

RSZYD18187977_A_saliva.metaspades.bin.14
RDPYD18089315_A_tongue.metaspades.bin.99
RSZYD18078715_A_saliva.metaspades.bin.28
RSZYD18187624_A_saliva.metaspades.bin.36
RSZYD18187425_A_saliva.metaspades.bin.14
RSZYD18078116_A_saliva.metaspades.bin.38
RSZYD18187390_A_saliva.metaspades.bin.24
RDPYD18089232_A_tongue.metaspades.bin.5
RSZYD18078546_A_saliva.metaspades.bin.49
RDPYD18300076_A_saliva.metaspades.bin.31
RSZYD18078717_A_saliva.metaspades.bin.35
RSZYD18078274_A_saliva.metaspades.bin.6
RSZYD18187966_A_saliva.metaspades.bin.2
RDPYD18300309_A_saliva.metaspades.bin.81
RSZYD18078701_A_saliva.metaspades.bin.25
RSZYD18187206_A_saliva.metaspades.bin.15
RDPYD18300297_A_saliva.metaspades.bin.2
RSZYD18187858_A_saliva.metaspades.bin.9
RSZYD18078785_A_saliva.metaspades.bin.23
RSZYD18078535_A_saliva.metaspades.bin.1
RSZYD18078771_A_saliva.metaspades.bin.12
RDPYD18300063_A_saliva.metaspades.bin.34
RSZYD18078688_A_saliva.metaspades.bin.48
RSZYD18078694_A_saliva.metaspades.bin.15
RSZYD18187603_A_saliva.metaspades.bin.2
RSZYD18078345_A_saliva.metaspades.bin.6
RSZYD18187495_A_saliva.metaspades.bin.13
RSZYD18078483_A_saliva.metaspades.bin.13
RSZYD18078130_A_saliva.metaspades.bin.15
MAG in family of *Ca.* bgiplasma
0%
25%
50%
75%
100%
Proportion of COGs
Metabolism
Energy production and conversion
Carbohydrate transport and metabolism
Amino acid transport and metabolism
Nucleotide transport and metabolism
Coenzyme transport and metabolism
Lipid transport and metabolism
Inorganic ion transport and metabolism
Secondary metabolites biosynthesis, transport and catabolism
Cellular processes and signaling
Cell cycle control, cell division, chromosome partitioning
Defense mechanisms
Signal transduction mechanisms
Cell wall/membrane/envelope biogenesis
Cell motility
Cytoskeleton
Intracellular trafficking, secretion, and vesicular transport
Posttranslational modification, protein turnover, chaperones
Information storage and processing
Translation, ribosomal structure and biogenesis
Transcription
Replication, recombination and repair
Poorly characterized
Function unknown
No hit
