# Supplementary material for: Over 50,000 Metagenomically Assembled Draft Genomes for the Human Oral Microbiome Reveal New Taxa
Source: Genomics Proteomics Bioinformatics. 2021 Sep 4;20(2):246–59. doi: 10.1016/j.gpb.2021.05.001 (PMC9684161; doi:10.1016/j.gpb.2021.05.001)
Supplement: Supplementary Figure S5 — The pangenome genetic diversity The gene number rarefaction curve for SGBs in the eight most prevalent genus. X-axis is the ratio of randomly selected genomes divide total genome numbers in each SGB. Solid lines are only included public reference genomes in SGBs. Dashed lines are included both public reference genome and our reconstructed MAGs in SGBs. Curves are colored by SGBs. [file mmc5.pdf]

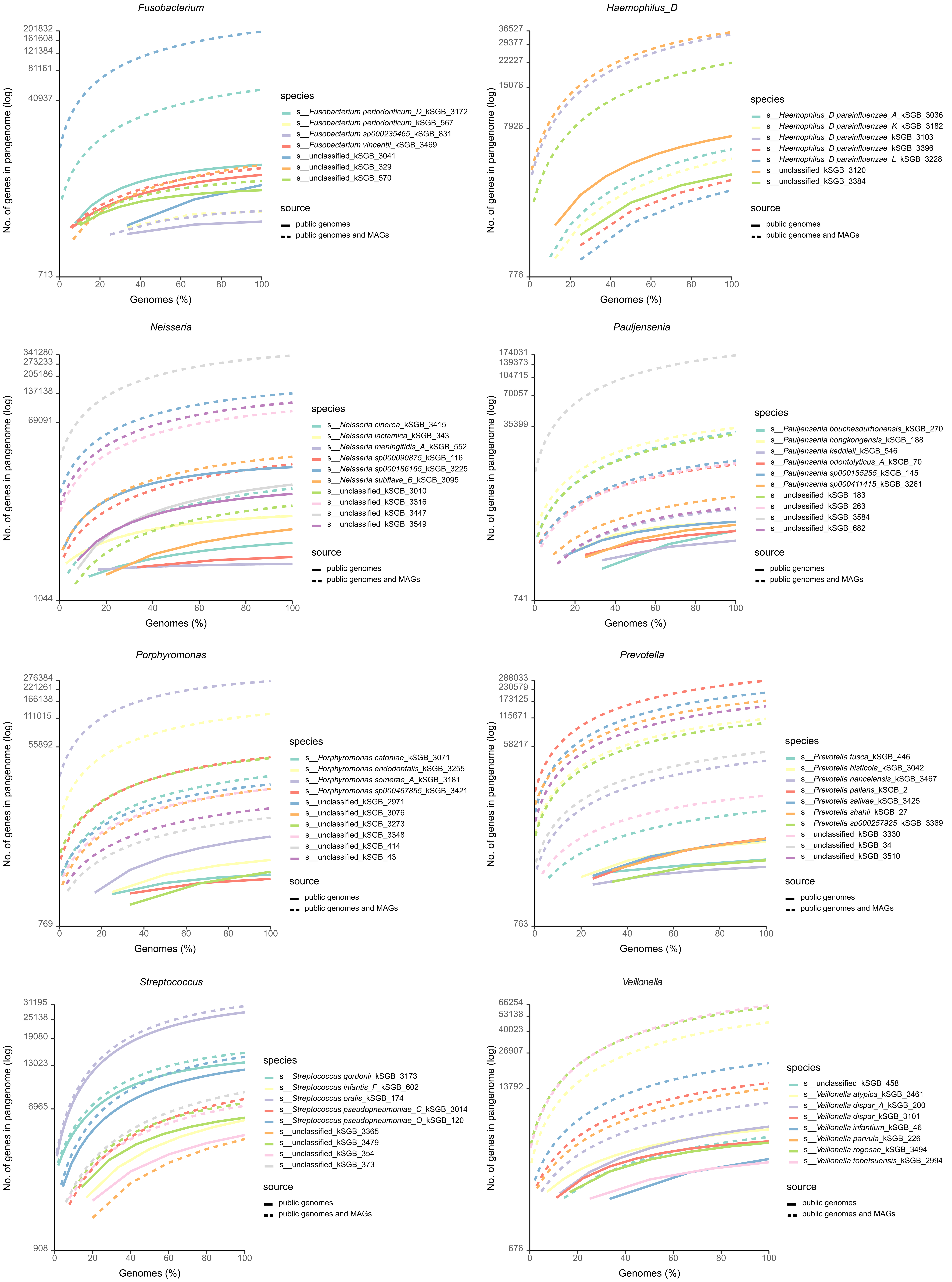
Fusobacterium
No. of genes in pangenome (log)
201832
161608
121384
81161
40937
713
Genomes (%)
0
20
40
60
80
100
species
s__Fusobacterium periodonticum_D_kSGB_3172
s__Fusobacterium periodonticum_kSGB_567
s__Fusobacterium sp000235465_kSGB_831
s__Fusobacterium vincentii_kSGB_3469
s__unclassified_kSGB_3041
s__unclassified_kSGB_329
s__unclassified_kSGB_570
source
public genomes
public genomes and MAGs
Haemophilus_D
36527
29377
22227
15076
7926
776
s__Haemophilus_D parainfluenzae_A_kSGB_3036
s__Haemophilus_D parainfluenzae_K_kSGB_3182
s__Haemophilus_D parainfluenzae_kSGB_3103
s__Haemophilus_D parainfluenzae_kSGB_3396
s__Haemophilus_D parainfluenzae_L_kSGB_3228
s__unclassified_kSGB_3120
s__unclassified_kSGB_3384
Neisseria
341280
273233
205186
137138
69091
1044
s__Neisseria cinerea_kSGB_3415
s__Neisseria lactamica_kSGB_343
s__Neisseria meningitidis_A_kSGB_552
s__Neisseria sp000090875_kSGB_116
s__Neisseria sp000186165_kSGB_3225
s__Neisseria subflava_B_kSGB_3095
s__unclassified_kSGB_3010
s__unclassified_kSGB_3316
s__unclassified_kSGB_3447
s__unclassified_kSGB_3549
Pauljensenia
174031
139373
104715
70057
35399
741
s__Pauljensenia bouchesdurhonensis_kSGB_270
s__Pauljensenia hongkongensis_kSGB_188
s__Pauljensenia keddieii_kSGB_546
s__Pauljensenia odontolyticus_A_kSGB_70
s__Pauljensenia sp000185285_kSGB_145
s__Pauljensenia sp000411415_kSGB_3261
s__unclassified_kSGB_183
s__unclassified_kSGB_263
s__unclassified_kSGB_3584
s__unclassified_kSGB_682
Porphyromonas
276384
221261
166138
111015
55892
769
s__Porphyromonas catoniae_kSGB_3071
s__Porphyromonas endodontalis_kSGB_3255
s__Porphyromonas somerae_A_kSGB_3181
s__Porphyromonas sp000467855_kSGB_3421
s__unclassified_kSGB_2971
s__unclassified_kSGB_3076
s__unclassified_kSGB_3273
s__unclassified_kSGB_3348
s__unclassified_kSGB_414
s__unclassified_kSGB_43
Prevotella
288033
230579
173125
115671
58217
763
s__Prevotella fusca_kSGB_446
s__Prevotella histicola_kSGB_3042
s__Prevotella nanceiensis_kSGB_3467
s__Prevotella pallens_kSGB_2
s__Prevotella salivae_kSGB_3425
s__Prevotella shahii_kSGB_27
s__Prevotella sp000257925_kSGB_3369
s__unclassified_kSGB_3330
s__unclassified_kSGB_34
s__unclassified_kSGB_3510
Streptococcus
31195
25138
19080
13023
6965
908
s__Streptococcus gordonii_kSGB_3173
s__Streptococcus infantis_F_kSGB_602
s__Streptococcus oralis_kSGB_174
s__Streptococcus pseudopneumoniae_C_kSGB_3014
s__Streptococcus pseudopneumoniae_O_kSGB_120
s__unclassified_kSGB_3365
s__unclassified_kSGB_3479
s__unclassified_kSGB_354
s__unclassified_kSGB_373
Veillonella
66254
53138
40023
26907
13792
676
s__unclassified_kSGB_458
s__Veillonella atypica_kSGB_3461
s__Veillonella dispar_A_kSGB_200
s__Veillonella dispar_kSGB_3101
s__Veillonella infantium_kSGB_46
s__Veillonella parvula_kSGB_226
s__Veillonella rogosae_kSGB_3494
s__Veillonella tobetsuensis_kSGB_2994
