# Supplementary material for: Over 50,000 Metagenomically Assembled Draft Genomes for the Human Oral Microbiome Reveal New Taxa
Source: Genomics Proteomics Bioinformatics. 2021 Sep 4;20(2):246–59. doi: 10.1016/j.gpb.2021.05.001 (PMC9684161; doi:10.1016/j.gpb.2021.05.001)
Supplement: Supplementary Figure S7 — Disease markers according to the oral genomes A. SGBs association with RA. Left panel, the Manhattan plot shows metagenomic wise association of oral SGBs for RA. The species are ordered according to their phylogeny (bottom) and the association direction (left side of the specie name are negative and right side of specie name are positive). Each point is one SGB and point height indicates the FDR value correction for multiple hypothesis tests from a generalized linear model test between diseased and healthy species abundance after adjusting age, gender, and BMI. Right panel, SGBs association with RA. We select 40 large (> 10 genomes) and most importance oral SGBs for disease prediction using GBM. The species are order according to their partial spearman correlation adjusted age, gender, BMI, and GBM importance. The bar length indicated the FDR value between groups as described above. The dotted line indicates the FDR of 1%. The red square in bar is the sqrt GBM importance and share the same X-axis with FDR value. uSGBs are highlight in bold label text. B. SGBs association with CRC. FDR, false discovery rate. GBM, gradient boosting machine. [file mmc7.pdf]

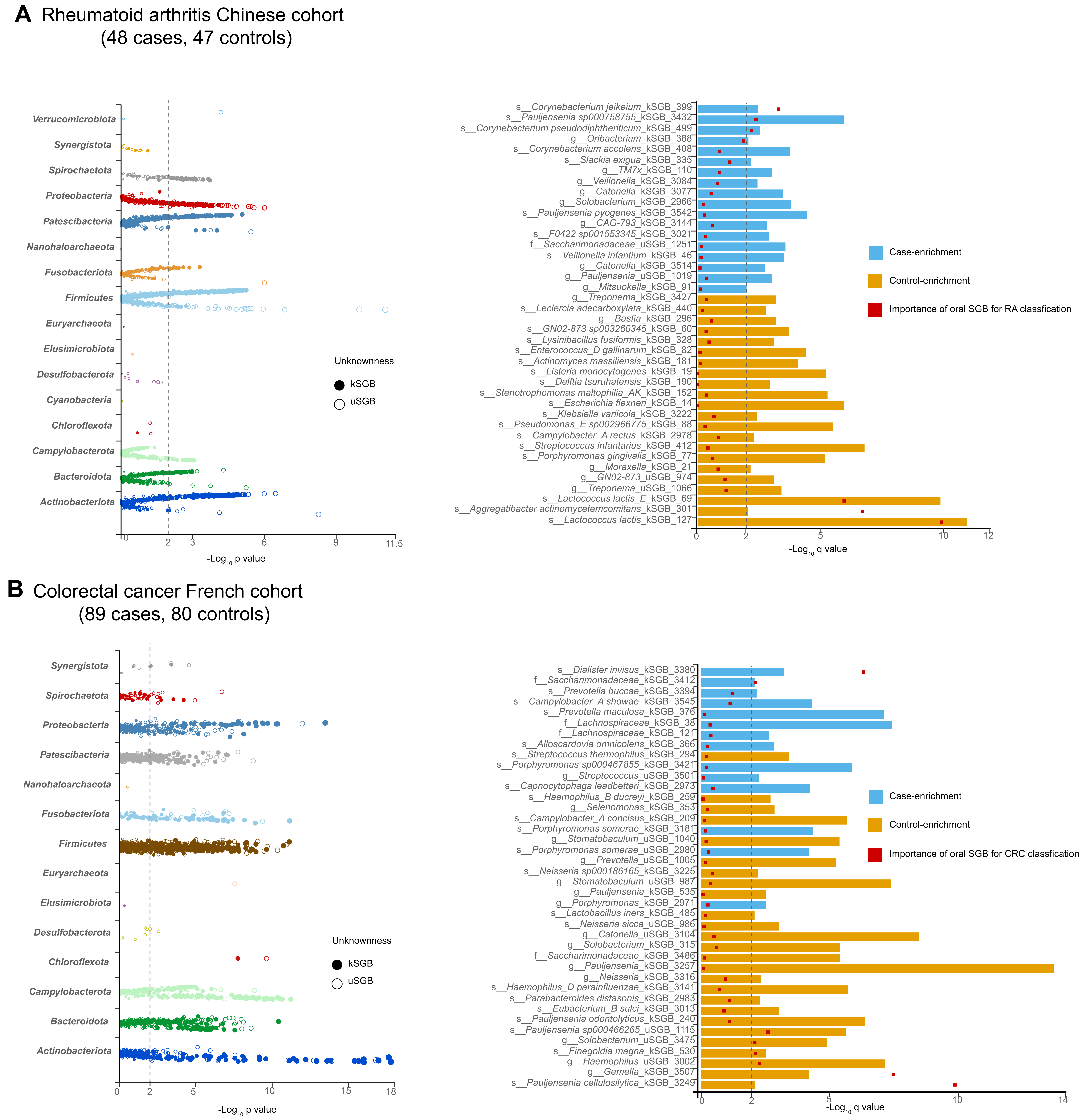
A Rheumatoid arthritis Chinese cohort
(48 cases, 47 controls)
Verrucomicrobiota
Synergistota
Spirochaetota
Proteobacteria
Patescibacteria
Nanohaloarchaeota
Fusobacteriota
Firmicutes
Euryarchaeota
Elusimicrobiota
Desulfobacterota
Cyanobacteria
Chloroflexota
Campylobacterota
Bacteroidota
Actinobacteriota
0
2
3
6
9
11.5
-Log10 p value
Unknownness
kSGB
uSGB
s__Corynebacterium jeikeium_kSGB_399
s__Pauljensenia sp000758755_kSGB_3432
s__Corynebacterium pseudodiphtheriticum_kSGB_499
g__Oribacterium_kSGB_388
s__Corynebacterium accolens_kSGB_408
s__Slackia exigua_kSGB_335
g__TM7x_kSGB_110
g__Veillonella_kSGB_3084
g__Catonella_kSGB_3077
g__Solobacterium_kSGB_2966
s__Pauljensenia pyogenes_kSGB_3542
g__CAG-793_kSGB_3144
s__F0422 sp001553345_kSGB_3021
f__Saccharimonadaceae_uSGB_1251
s__Veillonella infantium_kSGB_46
g__Catonella_kSGB_3514
g__Pauljensenia_uSGB_1019
g__Mitsuokella_kSGB_91
g__Treponema_kSGB_3427
s__Leclercia adecarboxylata_kSGB_440
g__Basfia_kSGB_296
s__GN02-873 sp003260345_kSGB_60
s__Lysinibacillus fusiformis_kSGB_328
s__Enterococcus_D gallinarum_kSGB_82
s__Actinomyces massiliensis_kSGB_181
s__Listeria monocytogenes_kSGB_19
s__Delftia tsuruhatensis_kSGB_190
s__Stenotrophomonas maltophilia_AK_kSGB_152
s__Escherichia flexneri_kSGB_14
s__Klebsiella variicola_kSGB_3222
s__Pseudomonas_E sp002966775_kSGB_88
s__Campylobacter_A rectus_kSGB_2978
s__Streptococcus infantarius_kSGB_412
s__Porphyromonas gingivalis_kSGB_77
g__Moraxella_kSGB_21
g__GN02-873_uSGB_974
g__Treponema_uSGB_1066
s__Lactococcus lactis_E_kSGB_69
s__Aggregatibacter actinomycetemcomitans_kSGB_301
s__Lactococcus lactis_kSGB_127
0
2
5
10
12
-Log10 q value
Case-enrichment
Control-enrichment
Importance of oral SGB for RA classfication
B Colorectal cancer French cohort
(89 cases, 80 controls)
Synergistota
Spirochaetota
Proteobacteria
Patescibacteria
Nanohaloarchaeota
Fusobacteriota
Firmicutes
Euryarchaeota
Elusimicrobiota
Desulfobacterota
Chloroflexota
Campylobacterota
Bacteroidota
Actinobacteriota
0
2
5
10
15
18
-Log10 p value
Unknownness
kSGB
uSGB
s__Dialister invisus_kSGB_3380
f__Saccharimonadaceae_kSGB_3412
s__Prevotella buccae_kSGB_3394
s__Campylobacter_A showae_kSGB_3545
s__Prevotella maculosa_kSGB_376
f__Lachnospiraceae_kSGB_38
f__Lachnospiraceae_kSGB_121
s__Alloscardovia omnicolens_kSGB_366
s__Streptococcus thermophilus_kSGB_294
s__Porphyromonas sp000467855_kSGB_3421
g__Streptococcus_uSGB_3501
s__Capnocytophaga leadbetteri_kSGB_2973
s__Haemophilus_B ducreyi_kSGB_259
g__Selenomonas_kSGB_353
s__Campylobacter_A concisus_kSGB_209
s__Porphyromonas somerae_kSGB_3181
g__Stomatobaculum_uSGB_1040
s__Porphyromonas somerae_uSGB_2980
g__Prevotella_uSGB_1005
s__Neisseria sp000186165_kSGB_3225
g__Stomatobaculum_uSGB_987
g__Pauljensenia_kSGB_535
g__Porphyromonas_kSGB_2971
s__Lactobacillus iners_kSGB_485
s__Neisseria sicca_uSGB_986
g__Catonella_uSGB_3104
g__Solobacterium_kSGB_315
f__Saccharimonadaceae_kSGB_3486
g__Pauljensenia_kSGB_3257
g__Neisseria_kSGB_3316
s__Haemophilus_D parainfluenzae_kSGB_3141
s__Parabacteroides distasonis_kSGB_2983
s__Eubacterium_B sulci_kSGB_3013
s__Pauljensenia odontolyticus_kSGB_240
s__Pauljensenia sp000466265_uSGB_1115
g__Solobacterium_uSGB_3475
s__Finegoldia magna_kSGB_530
g__Haemophilus_uSGB_3002
g__Gemella_kSGB_3507
s__Pauljensenia cellulosilytica_kSGB_3249
0
2
5
10
14
-Log10 q value
Case-enrichment
Control-enrichment
Importance of oral SGB for CRC classfication
